# Photobiomodulation improves the synapses and cognitive function and ameliorates epileptic seizure by inhibiting downregulation of Nlgn3

**DOI:** 10.1186/s13578-022-00949-6

**Published:** 2023-01-12

**Authors:** Namgue Hong, Hee Jung Kim, Keunsoo Kang, Ji On Park, Seyoung Mun, Hyung-Gun Kim, Bong Hui Kang, Phil-Sang Chung, Min Young Lee, Jin-Chul Ahn

**Affiliations:** 1 Medical Laser Research Center, Dankook University, Cheonan, Republic of Korea; 2 Department of Biomedical Science, College of Medicine, Dankook University, Cheonan, Republic of Korea; 3 Department of Physiology, College of Medicine, Dankook University, Cheonan, Republic of Korea; 4 Department of Microbiology, College of Science & Technology, Dankook University, Cheonan, Republic of Korea; 5 Department of Medicine, Graduate School of Dankook University, Dankook University, Cheonan, Republic of Korea; 6 Center for Bio-Medical Engineering Core Facility, Dankook University, Cheonan, Republic of Korea; 7 Department of Pharmacology, College of Medicine, Dankook University, Cheonan, Republic of Korea; 8 Department of Neurology, Dankook University Hospital, Dankook University, Cheonan, Republic of Korea; 9 Beckman Laser Institute Korea, Dankook University Hospital, Dankook University, Cheonan, Republic of Korea; 10 Department of Otolaryngology-Head & Neck Surgery, College of Medicine, Dankook University Hospital, Dankook University, Cheonan, Republic of Korea

**Keywords:** Temporal lobe epilepsy, Excitotoxicity, Near infrared light, Synapse, Neuroligin-3

## Abstract

**Background:**

Temporal lobe epilepsy (TLE) remains one of the most drug-resistant focal epilepsies. Glutamate excitotoxicity and neuroinflammation which leads to loss of synaptic proteins and neuronal death appear to represent a pathogen that characterizes the neurobiology of TLE. Photobiomodulation (PBM) is a rapidly growing therapy for the attenuation of neuronal degeneration harboring non-invasiveness benefits. However, the detailed effects of PBM on excitotoxicity or neuroinflammation remain unclear. We investigated whether tPBM exerts neuroprotective effects on hippocampal neurons in epilepsy mouse model by regulating synapse and synapse-related genes.

**Methods:**

In an in vitro study, we performed imaging analysis and western blot in primary hippocampal neurons from embryonic (E17) rat pups. In an in vivo study, RNA sequencing was performed to identify the gene regulatory by PBM. Histological stain and immunohistochemistry analyses were used to assess synaptic connections, neuroinflammation and neuronal survival. Behavioral tests were used to evaluate the effects of PBM on cognitive functions.

**Results:**

PBM was upregulated synaptic connections in an in vitro. In addition, it was confirmed that transcranial PBM reduced synaptic degeneration, neuronal apoptosis, and neuroinflammation in an in vivo. These effects of PBM were supported by RNA sequencing results showing the relation of PBM with gene regulatory networks of neuronal functions. Specifically, Nlgn3 showed increase after PBM and silencing the Nlgn3 reversed the positive effect of PBM in in vitro. Lastly, behavioral alterations including hypoactivity, anxiety and impaired memory were recovered along with the reduction of seizure score in PBM-treated mice.

**Conclusions:**

Our findings demonstrate that PBM attenuates epileptic excitotoxicity, neurodegeneration and cognitive decline induced by TLE through inhibition of the Nlgn3 gene decrease induced by excitotoxicity.

**Graphical Abstract:**

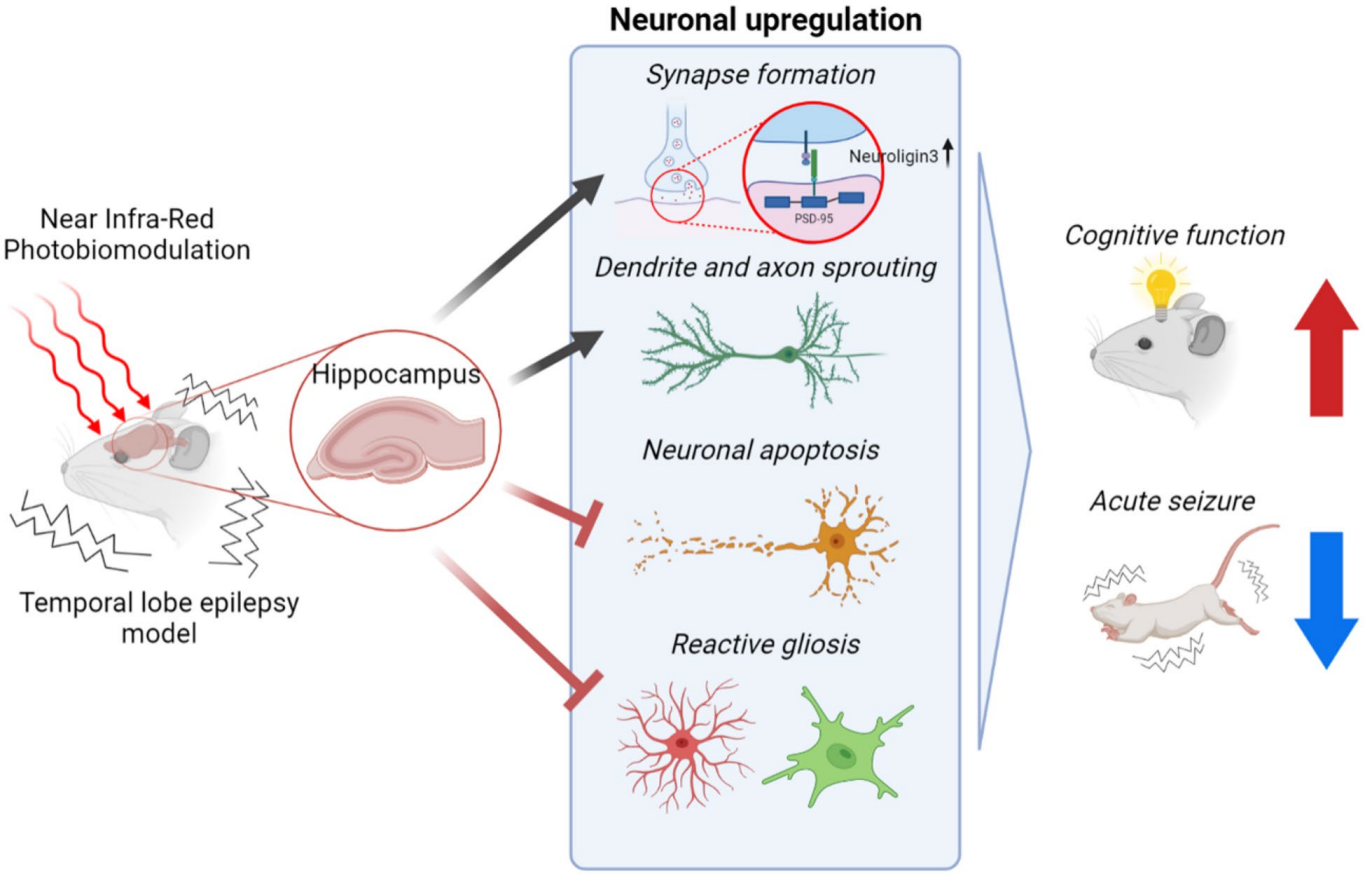

**Supplementary Information:**

The online version contains supplementary material available at 10.1186/s13578-022-00949-6.

## Background

Temporal lobe epilepsy (TLE) is one of neurological disorders associated with sequalae of excitotoxicity and is characterized by localization of seizure foci caused by abnormal hyperexcitation and synchronization of neuronal activity in the limbic system, entorhinal cortex and, particularly, the hippocampus. TLE-derived neurodegeneration impairs cognition and damages hippocampal circuitry, leading to progressive behavioral abnormalities [1, 2]. TLE remains one of the most drug-resistant focal epilepsies in the adult population; patients with mesial temporal sclerosis have the worst prognosis and as much as 11–42% can be seizure-free with appropriate treatment [3, 4]. How an initial brain insult elicits epilepsy remains debatable; however, glutamate excitotoxicity and neuroinflammation appear to represent a pathogen that characterizes the neurobiology of different brain disorders including epilepsy, leading to seizure-induced cell death, increased susceptibility to neuronal synchronization, and network alterations.

Among the animal models of epilepsy available for evaluating epileptogenic mechanisms, pilocarpine (PI)-induced status epilepticus (SE) is widely used because it mimics TLE in humans and represents a validated animal model for studying the onset and development of chronic epilepsy [5, 6]. This animal model confirmed the role of excitotoxicity and neuroinflammation as the underlying molecular events of seizure with the most vulnerable location among neural structures being the hippocampus [7, 8].

Hippocampal vulnerability to excitotoxicity has been widely studied. Excitotoxicity-induced hyperexcitation leads to the loss of synaptic proteins, dendritic spines, and dendritic branches preceding neuronal death [9–11]. Indeed, loss of synaptic proteins, neuronal death, and neuroinflammation correlate with the cognitive decline underlying neurodegeneration [1, 12, 13]. The hyperexcitation of excitatory neurons is induced by chemoconvulsants such as PI and kainic acid, which activate muscarinic acetylcholine receptors and glutamatergic receptors, respectively [14].

Neuroligin is a transmembrane cell adhesion protein that is localized at the postsynaptic membrane and also contributes to the maintenance of synapses with neurexin of presynaptic protein [15]. There are several neuroligin genes such as neuroligin-1 (Nlgn1), neuroligin-2 (Nlgn2), and neuroligin-3 (Nlgn3) between rodents and humans [15]. Neuroligins have been associated with synapse formation and maturation via their interactions with scaffolding proteins like PSD95 at excitatory postsynapses and gephyrin at inhibitory postsynapses [15]. Intriguingly, Nlgn3 is expressed at both types of synapses and an imbalance between these synapses leads to various behavioral abnormalities such as memory defeats and hyperactivity [16–18]. Therefore, finding the synaptic function of Nlgn3 in excitotoxicity and how it might relate to the behavioral and epileptic seizure symptoms is a long-cherished desire in neuroligin research.

Photobiomodulation (PBM), harboring non-invasiveness benefits, is a rapidly growing approach to many medical indications including retinal diseases, stroke, neuromuscular disorders, and mood disorders [19–24]. PBM can be utilized for a wide variety of processes that can benefit various brain disorders [20]. The research of PBM for the treatment of neurodegenerative diseases has increased over the last decade [20, 21, 25, 26]. PBM may be an alternative treatment for the prevention or attenuation of neuronal degeneration that does not induce biological side effects, which is a limitation of drugs that affect brain function. In most cases, however, the detailed effects of PBM on excitotoxicity induced by epilepsy and its mechanism remain unclear.

Here, using PI and kainic acid in in vitro and in vivo models of excitotoxicity, we defined the role of NIR PBM in synaptic connections and associated cognitive function. Using molecular analyses, we showed that PBM upregulated and protected synaptic connections and neuronal functions in an in vitro excitotoxicity model. Importantly, we provide evidence that transcranial PBM (tPBM) reduced synaptic degeneration, neuroinflammation, and neuronal apoptosis using animal models of excitotoxicity induced by epilepsy. These effects of PBM were supported by RNA sequencing results showing the relation of PBM with gene regulatory networks of neuronal functions. Specifically, Nlgn3 showed increase after PBM and silencing the Nlgn3 reversed the serial positive effect of PBM in in vitro. In addition, we have shown that the neuro-regulatory effects of PBM were accompanied by behavioral alterations including hypoactivity, anxiety and impaired working memory.

## Methods

### Animals

All animals (male C57BL/6 mice, 18–28 g, 7–8 weeks of age, and maternal SD rats, TP16) were bred and housed in the animal facility at 21 °C under 12-h light/12-h dark cycles (lights on at 6 a.m.) with access to food pellets and water ad libitum. All the experiments were performed in compliance with the National Institutes of Health guidelines for animal research.

### Primary culture of hippocampal neurons

**Fig. 1:**
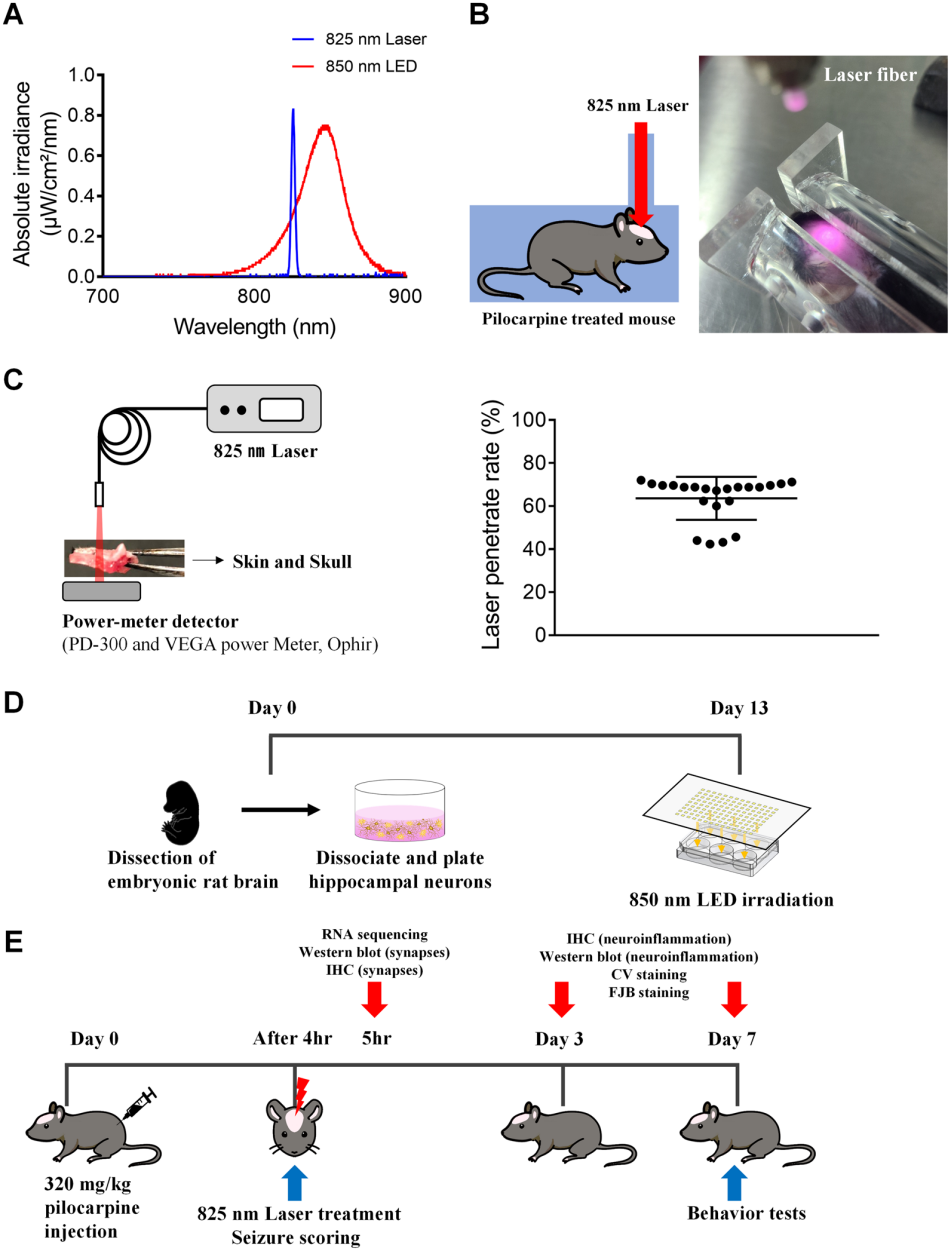
PBM irradiance and experimental schedule for in vivo and in vitro study. **A** Beam profiling of PBM using a spectrophotometer. An 825 nm laser with specific narrow wavelength was use to irradiate the SE mouse and an 850 nm LED with a wide range of wavelengths was use to irradiate the primary hippocampal neurons. **B** Experimental setup for treatment with tPBM. After the hair on the head was depilated, the mouse was placed in the mouse restrainer with a hole in the upper part to fix the movement, and then an 825 nm laser was used for irradiation for 380 s at a distance of 4 cm with a power of 40 mW/cm^2^. **C** Laser transmission ratio to skin and skull were measured by power-meter detector. **D** Rat hippocampal primary cultured neurons were irradiated by 850 nm LED on DIV 13. **E** 825 nm laser was irradiated at 4 h after PI injection, seizure scoring was performed on day 0, and behavior tests were performed on day 7. Then, brains were collected for RNA-sequencing, histology, IHC, western blotting, and biochemical analyses on days 0, 3, or 7

Rat hippocampal neurons were prepared from TP 17 SD rats (Fig. 1D). Fetuses were removed on embryonic day 17 from maternal rats anesthetized with 16.5% urethane. Hippocampi were dissected and placed in Ca^2+^- and Mg^2+^-free HEPES buffered Hank’s salt solution (pH 7.45). Cells were dissociated by trituration through a series of flame-narrowed Pasteur pipettes. Then, dissociated cells were plated at a density of 16,000 cells/well onto a 25-mm-round cover glass containing neurobasal medium with l-glutamine, 2% B27 supplement, 0.25% Glutamax I, and penicillin/streptomycin/amphotericin B (100 U/mL, 100 µg/mL, and 0.025 µg/mL, respectively). The cover glass was coated with Matrigel (0.2 mg/mL; BD Bioscience) for 1 h. Neurons were grown in a humidified atmosphere of 10% CO_2_ and 90% air (pH 7.4) at 37 °C and fed at 4-day intervals by replacing 75% of the media with a fresh media. All in vitro experiments consisted of six independent cultures.

Following the manufacturer’s instructions, transfection with small interfering RNA (siRNA) was initiated by adding a mixture (1:1) of Lipofectamine 2000 Reagent diluted in Opti‐MEM Medium (1:50) and the siRNA (abx925999, Abbexa), also diluted in Opti‐MEM Medium (1:50) for 5 min. Then, the cells were incubated with the siRNA‐Lipid complex for 24 h at 37 °C.

### PI-induced chemoconvulsant models

Male C57BL/6 mice 7–8 weeks of age were administered scopolamine methyl nitrate (intraperitoneally (i.p.); 1.5 mg/kg; Sigma-Aldrich) and terbutaline hemisulfate salt (i.p.; 1.5 mg/kg; Sigma-Aldrich) to block peripheral effects of PI and dilate the respiratory tract, respectively. PI hydrochloride (i.p.; Sigma-Aldrich) at 320 mg/kg was injected 30 min later (Fig. 1E). After continuous tonic clonic convulsive seizures were initiated, mice were placed at room temperature for 8 h and monitored with video recoding. Acute seizures were behaviorally scored using a modified Racine scale (Additional file 1: Videos S1–S6, stages: 0, no abnormality; 1, exploring, sniffing, and grooming ceased, becoming motionless; 2, forelimb and/or tail extension, appearance of rigid posture; 3, myoclonic jerks of the head and neck, with brief twitching movement, or repetitive movements with head bobbing; 4, forelimb clonus and partial rearing, or occasional rearing and falling; 5, forelimb clonus, continuous rearing and falling; and 6, tonic–clonic movements with loss of posture tone, often resulting in death) [27–29]. Only mice showing multiple stage 5 or above behavioral seizures were selected for further processing as status epilepticus.

### PBM irradiation

Irradiation was performed using an 825 nm laser with total irradiance at skin surface of 40 mW/cm^2^ and a total energy density of 15.2 J/cm^2^ for a 380 s exposure (Fig. 1A, B, Table 1). Mice were maintained motionless by restraint in the restrainer and the laser was manually applied at 4 cm from the skin of the shaved head. Laser transmission ratio to skin and skull was measured before the laser treatment (penetration rate of PBM; Fig. 1C), which was applied once 4 h after PI injection (Fig. 1E). Hippocampal primary neurons were exposed for 600 s to a 850 nm LED device with a power density of 16 mW/cm^2^ and a total energy density of 9.6 J/cm^2^ (Fig. 1A, Table 2). Cells were irradiated 30 min after the treatment with kainic acid on day in vitro (DIV) 13 in the dark, followed by a fixative 4 h after treatment with kainic acid (Fig. 1D). A powermeter (PD300 and VEGA, Ophir PD, Ophir Photonics) was used to measure the light source power density.

**Table 1:** Specifications for laser parameters

Irradiation parameters	
Central wavelength (nm)	825
Wavelength tolerance (nm)	± 10
Max output power (W)	1
Fiber core diameter (µm)	60
Mode	Continuous wave mode
Beam shape	Circular
Numerical aperture	0.14
Spectral width (FWHM, nm)	< 3.0

Treatment parameters	
Irradiance power at skin (mW/cm^2^)	40
Laser transmission ratio to skin and skull (%)	63.6
Distance to the head from the end of a fiber (cm)	4
Exposure duration (s)	380
Focused beam spot size (mm^2^)	14.01
Number of points irradiated	1
Radiant exposure at brain (J/cm^2^)	15.2

**Table 2:** Specifications for LED parameters

Irradiation parameters	
Central wavelength (nm)	850
Wavelength tolerance (nm)	± 10
Mode	Continuous wave mode
Voltage value (V)	29.5

Treatment parameters	
Irradiance power (mW/cm^2^)	16
Distance to LED from cell culture plate (cm)	4
Exposure duration (s)	600
Number of points irradiated	1
Radiant exposure at neurons (J/cm^2^)	9.6

### Neuronal death based on Nissl staining analysis

After PI-induced seizures, mice were anesthetized 3 and 7 days later with ethyl ether and transcardially perfused with cold saline using a pump and fixed in 4% paraformaldehyde in 0.1 M phosphate buffer, pH 7.4. The brains were removed from the skulls, fixed for at least 48 h, then cryoprotected in 30% sucrose in phosphate-buffered saline. Sequential coronal sections (18-µm-thick) through the hippocampus were prepared using a cryocut microtome (CM3050S, Leica). The tissues were mounted on gelatin-coated slides overnight before use. After dehydration in a graded alcohol series, hippocampal sections were stained for 20 min with pre-warmed 0.3% CV solution at room temperature. After destaining with 95% ethanol and 0.3% glacial acetic acid solution, the sections were dehydrated using 100% ethanol followed by 100% xylene. Then, the sections were mounted with DPX. Unbiased cell counting was obtained in the CA1, CA3, and hilus. Only cells displaying Nissl-stained cytoplasm with a nucleus top completely within the section were counted. Cells were included if they were partly or entirely within the dissector frame and did not cross the exclusion lines. The counted average range for CA1, CA3, and hilus was 200 μm, 200 μm, and 1 mm^2^, respectively.

### Neurodegeneration based on FJB staining

Coronal sections (18-µm-thick) were stained with FJB solution. The tissues were mounted on gelatin-coated slides overnight before use. After dehydration in a graded alcohol series, hippocampal sections were incubated in 0.06% potassium permanganate solution for 10 min. Next, brain sections were stained with 0.0004% FJB solution containing 0.1% glacial acetic acid for 20 min at room temperature. Then, the sections were washed with distilled water, dried, and mounted with DPX. Unbiased cell counting was also obtained in the CA1, CA3, and hilus and counted average range was 200 μm, 200 μm, and 1 mm^2^, respectively.

### Immunocytochemistry and immunohistochemistry

Primary hippocampal neurons were fixed with chilled methanol for 8 min at − 20 °C. Hippocampal tissue sections and primary neurons were permeabilized with 0.3% Triton X-100 for 5 min. The tissues and cells were blocked with 10% bovine serum albumin (BSA) and incubated overnight at 4 °C with the following primary antibodies: mouse anti-MAP2 (Sigma-Aldrich, M9942), rabbit anti-PSD95 (Abcam, ab18258), rabbit anti-Synaptophysin (Abcam, ab32127), mouse anti-GFAP (Millipore, MAB360), rat anti-CD11b (Abcam, ab8878), mouse anti-Nlgn3 (Santa Cruz, SC-271880). After treatment with primary antibodies, the tissues and cells were incubated with secondary antibodies conjugated with Alexa Fluor 488-conjugated anti-rabbit IgG (ThermoFisher, A11008), Alexa Fluor 555-conjugated anti-mouse IgG (ThermoFisher, A21422), or Alexa Fluor 488-conjugated anti-rat IgG (ThermoFisher, A11006) for 1.5 h at room temperature. Immunostained tissues and cells were mounted with VECTASHIELD Antifade mounting medium with DAPI (H-1200; Vector Laboratories). Alexa Fluor 488 (excitation, 488 nm; emission, 520 nm) and Alexa Fluor 555 (excitation, 561 nm; emission, 568 nm)-labeled tissues and cells were imaged using a confocal microscope (FV3000, Olympus).

### Western blot analysis

Proteins of primary cultured neurons and homogenized hippocampal tissues were extracted in RIPA buffer (ThermoFisher) supplemented with a protease and phosphatase inhibitor cocktail (ThermoFisher). After centrifugation (4 °C, 13,000 rpm, 15 min), the supernatant was collected into a new 1.5 mL tube and stored at − 80 °C. Total proteins (15–30 μg) were resolved on 10–15% sodium dodecyl sulfate polyacrylamide gel electrophoresis (SDS-PAGE), and electrotransferred with transfer buffer to polyvinylidene fluoride transfer membrane (Bio-Rad). Then, the membranes were blocked in TBST (10 mM Tris- HCl, pH 7.4, 150 mM NaCl, 0.1% Tween 20) containing 5% BSA and incubated with the appropriate primary and secondary antibodies. PSD-95, MAP2, GFAP, Iba1 and Nlgn3 were detected in the membranes using anti-PSD95 (Abcam, ab18258), anti-MAP2 (Sigma-Aldrich, M9942), anti-GFAP (Millipore, MAB360), anti-Iba1 (Invitrogen, PA5-27436), and anti-Nlgn3 (Santa Cruz, SC-271880), respectively. β-Actin antibody (Sigma-Aldrich, A1978) was used as the standard. Protein bands were detected using the Clarity™ Western ECL Substrate (Bio-Rad). Bands were captured using Image Lab 6.0.1 (Bio-Rad). Densitometry analyses are presented as the ratio of protein to β-actin protein, which was compared with the controls and normalized.

### Transmission electron microscopy (TEM)

Hippocampi obtained from mouse brains were immediately fixed in 2% glutaraldehyde and 2% paraformaldehyde in 0.1 M phosphate buffer (pH 7.4) for 2 h at 4 °C. Following washes in deuterated H_2_O_2_, the tissues were dehydrated in ethanol and propylene oxide. The tissues were then embedded in EPON epoxy resin (Embed 812, Nadic methyl anhydride, poly Bed 812, dodecenylsuccinic anhydride, and dimethylaminomethyl phenol, Electron Microscopy Polysciences, USA). Ultrathin sections (65 nm) were obtained with a model ultramicrotome (RMC MT-XL) and collected on 100 mesh copper grids. The sections were imaged by TEM at 80 kV (JEOL, Japan).

### Microscopic analysis and quantification

Primary hippocampal neurons were imaged using the confocal microscope (FV3000, Olympus) and observed with 20× and 60× objective lenses. All multiple optical sections spanning 10 µm in the z-dimension were collected (1-µm steps) and the optical sections were combined through the z-axis into a compressed z-stack; 3–4 images from randomly selected fields were taken. The PSD95 puncta was counted in primary dendrites within proximal dendrites [30]. The puncta counting algorithm was performed using ImageJ software to quantify PSD95-positive puncta as previously described [31, 32]. Briefly, the maximum z-projection PSD95 image was applied with the maximum z-projection MAP2 image using a threshold set of one standard deviation (SD) above the image mean. This 1-bit created projection was used as a mask with the PSD95 maximum z-projection. Structures between 8 and 80 pixels (approximately 0.4–4.0 μm in diameter) were counted as PSD95. The structures were then dilated on the MAP2 maximum z-projection for visualization. The PSD95 puncta counts were presented as the mean ± standard error of the mean (SEM), where *n* is the number of dendrites from six different cultures. The 20× objective MAP2 confocal images were analyzed to evaluate dendritic morphological changes in primary hippocampal neurons using Metamorph software (Molecular Devices).

### Detection of cell viability based on CCK-8 assay

Protection against cell death was assayed using the CCK-8 kit (Dojindo). Primary hippocampal neurons (10,000 cells/well) were plated in 96-well plates and incubated for 24 h in 100 μL DMEM medium. Cells were seeded in triplicate for each treatment group. Kainic acid (150 μM) was added to the cells 30 min after, followed by 850 nm LED. Cells were incubated for an additional 24 h. Next, 10 μL CCK-8 solution was added to each well and the cell culture plate was incubated for 1–4 h. The absorbance at 450 nm was detected using a plate reader. Blank wells (culture media and CCK) and control wells (untreated cells, culture media, and CCK) were also detected.

### RNA-sequencing data analysis

The quality of sequenced reads (paired-end) was estimated using FastQC (https://www.bioinformatics.babraham.ac.uk/projects/fastqc/) and the sequenced reads were trimmed using Trim Galore (https://www.bioinformatics.babraham.ac.uk/projects/trim_galore/) with default settings. Trimmed reads were mapped to the mouse reference genome (mm10) using STAR [33] with default settings. Normalized expression levels of genes were calculated as transcripts per million (TPM) values using StringTie [34], and differentially expressed transcripts (adjusted p-value cutoff < 0.05 and |log_2_ fold-change| > 1) were identified using DESeq2 [35] after correcting potential batch-effects using RUVSeq [36]. The principal component analysis (PCA) plot was drawn using NetworkAnalyst [37], with the log2-transformed raw read count metric after removing the lowly expressed genes (raw read count < 4).

### Gene ontology analysis

Gene ontology analysis was performed with the Metascape application (https://metascape.org/gp/index.html#/main/step1) [38]. Upregulated or downregulated differentially expressed transcripts were used as input.

### Behavioral tests

Seven days after PI injection, mice were subjected to behavioral tests in the following order: OFT, novel object recognition test, and Y-maze spontaneous alternation. All behavioral tests occurred during the light phase. Mice were allowed to acclimate to each behavioral test once per day, for 3 days, 1 week before the behavioral experiment. The experiment was only performed if no motor seizure was observed for at least 1 h before the test. Prior to behavioral tests, mice were acclimated to the procedure room for at least 1 h. Cages were then moved to an adjacent room and mice were transported to the procedure room for tests one at a time. After tests were completed, mice were returned to their home cage and the maze was cleaned using a 70% EtOH solution between trials. All behavior tests were recorded using a mounted video camera and analyzed with EthovisionXT 12 software (Noldus Information Technology). After behavioral tests, mice were used in other experiments including immunohistochemistry, histology, and western blot.

#### Open field test

Locomotor and explorative activities and anxiety-like behavior were measured using the OFT, which was conducted in an opaque-sided box (measuring 50 cm long × 50 cm wide × 50 cm high) under indirect low illumination. Mice were individually placed in the center of the box and allowed to explore the maze for 5 min. Total distance traveled and number of line crossings were recorded for analysis of locomotor and explorative activities. Time spent in the center zone was recorded with percentage time spent in the center zone/(center + peripheral side zone) of the OFT used as a measure of anxiety-like behavior.

#### Novel object recognition test

The novel object recognition test was used to analyze the effects of tPBM on recognition memory. During habituation, all mice were introduced into an empty opaque-sided box (measuring 50 cm long × 50 cm wide × 50 cm high) for 10 min on the first day of testing. On the next day (training), the mice were exposed to two identical objects placed in the arena for 10 min. On test day (24 h later), the mice were allowed to explore one of the familiar objects and a novel object for 10 min. The time spent exploring the two objects was scored by the blinded observer using video-recorded sessions. Exploration was defined as pointing the head toward an object at a distance < 2 cm from the object. The exploration time was defined as the percentage of time that mice spent exploring familiar or novel objects with respect to the total exploration time.

#### Y maze spontaneous alternation

Working memory was assessed using the Y-maze spontaneous alternation test. The Y-maze apparatus consisted of three arms made of white plastic joined in the middle to form a “Y” shape. The diagonal walls of the identical arms were 15-cm-high from the ground, allowing the mouse to see distal spatial landmarks. This ethologically relevant test is based on the rodents’ innate curiosity to explore novel areas and presents no negative or positive stimulators and very little stress for the mice. The Y maze design was based on published protocols with modifications to adapt the system to mice. Briefly, mice were placed into the center of the maze and allowed to explore the maze for 3 days (habituation). The next day, mice were returned to the Y maze by placing them in the center of the maze. Then, the mice were allowed to freely explore all three arms of the maze for 5 min (test trial). Due to habituation, the maze was likely to be less stressful during the test trial. The recording of entry to each arm during trials was considered spontaneous alternation. Mice were subjected to a three-arm Y maze for 10 min with all three arms open. The number and the sequence of arms entered were recorded. The dependent variables were activity, defined as the number of arms entered, and percent alternation, calculated as the number of alternations (entries into three different arms consecutively) divided by the total possible alternations (i.e., the number of arms entered minus 2) and multiplied by 100.

### Statistics

Statistical analyses were performed using Prism 7.0 (GraphPad). All experimental data are presented as means ± SEM. A P value less than 0.05 was considered significant. Detailed statistical information for all Figures is described in Table 3.

**Table 3:** Statistical analysis of entire figures

Figure no.	Condition	Gaussian distribution	Column analyses	ANOVA value	ANOVA P value	P value
Figure 2B		SW	ANOVA	F = 11.21	P < 0.0001	**p = 0.0052
						***p = 0.0001
Figure 2C		AP	ANOVA	F = 22.92	P < 0.0001	****p < 0.0001
						***p = 0.0001
Figure 2D			KW	KW = 53.74	P < 0.0001	****p < 0.0001
						***p = 0.0009
Figure 2E			KW	KW = 45.29	P < 0.0001	****p < 0.0001
Figure 2F			KW	KW = 28.1	P < 0.0001	***p = 0.0004
						*p = 0.0129
Figure 2G			KW	KW = 72.75	P < 0.0001	****p < 0.0001
Figure 2H			KW	KW = 46.67	P < 0.0001	***p = 0.0005
						****p < 0.0001
Figure 2I		SW	ANOVA	F = 29.84	P < 0.0001	**p = 0.0095
						*p = 0.0186
						****p < 0.0001
Figure 3B			2way ANOVA	Row F = 32.41 Column F = 7.594	P < 0.0001 P = 0.0218	*p = 0.0261
Figure 4B			KW	KW = 29.81	P < 0.0001	****p < 0.0001
Figure 4C		SW	ANOVA	F = 22.54	P < 0.0001	****p < 0.0001
Figure 4D		AP	ANOVA	F = 31.49	P < 0.0001	****p < 0.0001
						***p = 0.0009
Figure 5B	D + 3	AP	Unpaired t-test			***p = 0.0001
	D + 7					****p < 0.0001
Figure 5C	D + 3	KS	Unpaired t-test			****p < 0.0001
	D + 7	AP				****p < 0.0001
Figure 5D	D + 3		MW	MWU = 1		****p < 0.0001
	D + 7			MWU = 60		****p < 0.0001
Figure 6B	CA1	SW	ANOVA	F = 27.19	P < 0.0001	***p = 0.0004
						^####^p < 0.0001
	CA3			F = 23.19	P < 0.0001	***p = 0.0008
						^###^p = 0.0001
	Hilus			F = 22.79	P < 0.0001	***p = 0.0004
						###p = 0.0003
Figure 6C	CA1	SW	ANOVA	F = 4.547	P = 0.0339	#p = 0.0467
	CA3			F = 9.805	P = 0.003	*p = 0.025
						^##^p = 0.0042
	Hilus			F = 34.49	P < 0.0001	***p = 0.0002
						^####^p < 0.0001
Figure 6D	GFAP		MW	MWU = 0		***p = 0.0006
	Iba1	SW	Unpaired t-test			****p < 0.0001
Figure 6E		AP	Unpaired t-test			****p < 0.0001
Figure 6F		AP	Unpaired t-test			**p = 0.0014
Figure 7C		SW	ANOVA	F = 114.6	P < 0.0001	****p < 0.0001
						***p = 0.0004
Figure 7E		AP	ANOVA	F = 6.893	P = 0.0008	**p = 0.0012
						*p = 0.0288
Figure 7G		AP	ANOVA	F = 14.42	P < 0.0001	*p = 0.0195
						****p < 0.0001
Figure 7H		AP	ANOVA	F = 10.44	P < 0.0001	**p = 0.0039
						****p < 0.0001
Figure 7J		SW	ANOVA	F = 8.665	P = 0.0012	**p = 0.0015
						*p = 0.0235
Figure 8B		AP	ANOVA	F = 11.9	P < 0.0001	*p = 0.0398
						**p = 0.0054
						^##^p = 0.0018
						***p = 0.0001
Figure 8C		SW	ANOVA	F = 7.006	P < 0.0001	*p = 0.0111
						*p = 0.0401
						**p = 0.0094
Figure 8D		SW	ANOVA	F = 8.197	P < 0.0001	**p = 0.0015
						*p = 0.013
						^##^p = 0.0093
Figure 8F		SW	ANOVA	F = 14.2	P < 0.0001	**p = 0.002
						****p < 0.0001
Figure 9B	PI		KW		P < 0.0001	**p = 0.0064
						***p = 0.0001
						****p < 0.0001
	PI + tPBM					^#^p = 0.0287
						^##^p = 0.0047
						^###^p = 0.0002
						^####^p < 0.0001
Figure 9C			MW	U = 137		**p = 0.0015
Figure 9D			Chi-Square	X^2^ = 3.0647		*p = 0.08
Figure 9F		AP	ANOVA	F = 24.04	P < 0.0001	****p < 0.0001
						**p = 0.0053
Figure 9G		AP	ANOVA	F = 21.84	P < 0.0001	****p < 0.0001
						*p = 0.035
Figure 9H			KW		P = 0.0003	***p = 0.0005
						*p = 0.014
Figure 9J		KS	ANOVA	F = 7.975	P = 0.004	**p = 0.0032
Figure 9L		AP	ANOVA	F = 13.43	P = 0.0004	***p = 0.0004
						*p = 0.012

*AP* D'Agostino & Pearson, *SW* Shapiro–Wilk, *KS* Kolmogorov–Smirnov, *ANOVA* one-way analysis of variance with Bonferroni post-hoc test, *KW* Kruskal–Wallis one-way analysis of variance with Dunn’s post-hoc test, *MW* two-tailed Mann–Whitney U test

*p < 0.05, **p < 0.01, ***p < 0.001, ****p < 0.0001, ^#^p < 0.05, ^##^p < 0.01, ^###^p < 0.001, ^####^p < 0.0001 relative to KA or PI

## Results

### Neuroregulatory effects of PBM against kainic acid-induced excitotoxicity on cultured hippocampal neurons

**Fig. 2:**
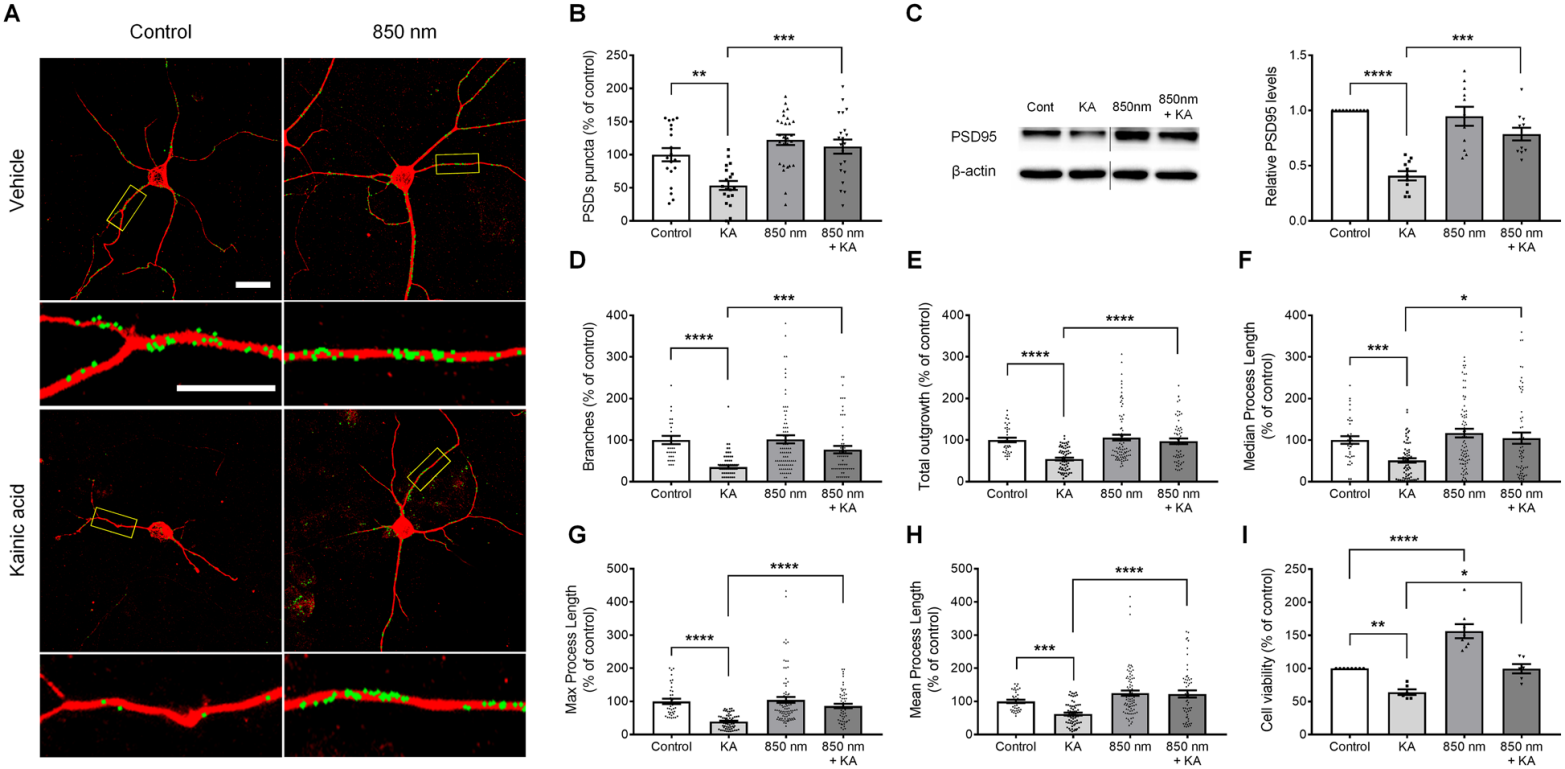
Kainic acid-induced synaptic loss and reduced dendritic morphology were reversed by PBM in cultured hippocampal neurons. **A** Representative images of PSD puncta analysis using the image processing algorithm showed that 850 nm PBM protected PSD puncta against kainic acid-induced PSD loss. PSD95-labeled green fluorescent puncta superimposed on MAP2 fluorescence (red) acquired before and 4 h after treatment with 150 µM kainic acid, and following 850 nm PBM 30 min after treatment with kainic acid. Confocal images were analyzed using ImageJ software. Labeled PSDs were dilated and overlaid on the MAP2 image for viewing purposes. The scale bars indicate 20 µm. Counting preceded dilation and labeling, so PSDs in close proximity were counted individually. The insets are enlarged images of the boxed region. The scale bars indicate 10 µm. **B** Bar graph shows significant changes in PSD puncta following treatment with 850 nm PBM in the absence (control) or presence of 150 µM kainic acid as indicated. **C** PSD95 of hippocampal primary cultured neurons was analyzed by western blotting with anti-PSD95 antibody. Full-length blots/gels are presented in Additional file 1: Fig. S1. In the bar graph, quantification of the normalized band intensities (ratio of PSD95 protein to β-actin) indicated that PSD95 significantly increased in the presence of 850 nm PBM. These lanes were run on the same gel but were noncontiguous. **D**–**H** Quantification of branches, total dendrite outgrowth, median process length, maximum process length, and mean process length per neuron based on MAP2 signals. Neurons in the 850 nm PBM group showed significantly longer dendrites. **I** Cell viability in primary hippocampal cultured neurons under control and 850 nm PBM condition groups were determined by the CCK-8 assay. 850 nm PBM increased cell viability after kainic acid-induced neuronal death. Data are expressed as the mean ± SEM; *p < 0.05, **p < 0.01, ***p < 0.001, ****p < 0.0001 relative to each control group (ANOVA with the Bonferroni test)

**Fig. 3:**
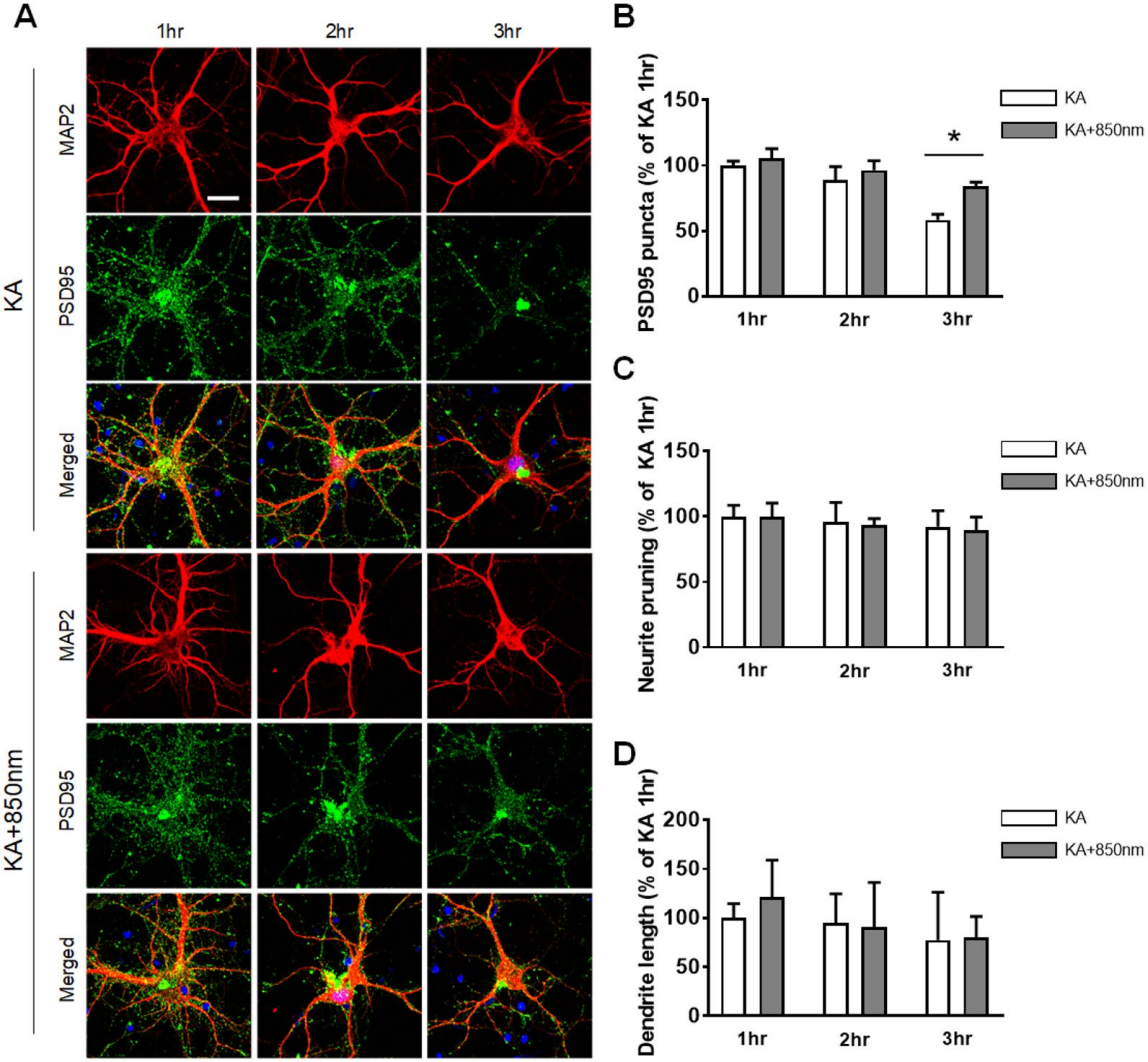
PBM affects the number of synapses prior to kainic acid induced morphological changes of dendrites and neuronal population. **A** Representative images of PSD95 and MAP2 at 1, 2, and 3 h showed that 850 nm PBM protected PSD95 puncta against kainic acid-induced PSD loss after 3 h. The scale bars indicate 20 µm. **B** Bar graph shows significant changes in PSD puncta following treatment with 850 nm PBM at 3 h in presence of kainic acid as indicated. Data are expressed as the mean ± SEM; *p < 0.05 relative to 3 h kainic acid (two-way ANOVA with the Bonferroni test). **C**, **D** Quantification of neurite pruning and dendrite length per neuron based on MAP2 signals. There are no significantly difference after 850 nm PBM treated

Kainic acid exposure induces synaptic loss, changes in dendritic morphologies, and neuronal apoptosis [39–44]. Overactivation of glutamate receptors by kainic acid leads to the membrane depolarization of neurons, inducing calcium ion (Ca^2+^) and subsequently triggering excitotoxic synapse loss and a cascade of events leading to neuronal death. We developed an imaging-based method to quantify the numbers of synaptic puncta on hippocampal neurons [31, 32]. In Fig. 2A, synaptic sites of the hippocampal neurons that were co-localized with PSD95 and MAP2 were identified. After treatment with kainic acid for 4 h, there was a significant decrease in synaptic sites by 53.6 ± 6.78% (n = 19) compared with the control (100 ± 9.95%, n = 19). Next, we determined whether PBM has synaptic-modulation effects against kainic acid-induced synapse loss. Cultured rat hippocampal neurons were treated with PBM at 850 nm wavelength and 6 Joules (10 mW/cm^2^, 10 min) for 30 min after kainic acid treatment. PBM significantly increased PSD puncta (112.25 ± 10.65%, n = 21) compared with that in kainic acid-induced synapse loss (Fig. 2B), showing that PBM inhibited kainic acid-induced synapse loss. These results were confirmed by western blotting using the cell lysates of hippocampal neurons (Additional file 1: Fig. S1). PSD95 expression was significantly decreased at 4 h after kainic acid treatment compared to the control (no kainic acid) when normalized to total β-actin (0.41 ± 0.04, n = 11), and was significantly increased after treatment with PBM and kainic acid compared to kainic acid only (0.79 ± 0.06, n = 11; Fig. 2C). Next, we determined whether PBM can regulate the morphological changes of neuronal dendrites in primary cultured hippocampal neurons. Our results showed that PBM increased the branches (76.65 ± 8.99%, n = 56), total outgrowth (96.85 ± 6.83%, n = 56), median process length (104.39 ± 13.37%, n = 56), maximum process length (86.38 ± 6.54%, n = 56), and mean process length (122.4 ± 10.76%, n = 56) of neuronal dendrites compared with kainic acid treatment by 34.78 ± 3.95% (n = 66), 53.85 ± 3.41% (n = 66), 50.61 ± 5.27% (n = 66), 39.09 ± 2.54% (n = 66), and 61.84 ± 4.17% (n = 66), respectively (Fig. 2D–H). These results suggest that PBM can increase kainic acid-induced excitotoxic morphological changes in neuronal dendrites. We have performed additional short duration (1 to 3 h) experiments and showed that in this time duration kainic acid degenerates the PSD95 but does not induce morphologic change of dendrites. With the application of PBM, the number of synaptic densities are maintained and relatively higher compared to kainic acid only without morphologic change of dendrites. There was statistical difference in density of PDS95 puncta between kainic acid and kainic acid with PBM group at 3 h time point (KA, 58.44 ± 4.26%, n = 8; KA + 850 nm, 83.88 ± 3.42%, n = 8). These results suggest that PBM action begins at a very early time point and provides evidence that PBM is affecting synaptic puncta densities (Fig. 3A, B).

We performed a cytotoxicity experiment with hippocampal neurons and determined whether they were affected by PBM. After 24 h of kainic acid treatment, cell viability was measured using the Cell Counting Kit-8 assay. As shown in Fig. 2I, cell viability was significantly decreased with kainic acid by 64.10 ± 4.33% (n = 6). By contrast, PBM significantly increased cell viability compared to the kainic acid only group by 99.52 ± 6.99% (n = 6). These results suggested that PBM regulated neurotoxicity induced by kainic acid, and indicated that kainic acid application decreased synaptic density, altered dendritic morphologies, and decreased the survival of neurons. These changes were reversed by PBM, suggesting the possibility of neuromodulation (upregulation) of neural structures in vitro, which was subsequently studied in vivo using a proper excitotoxicity model.

### tPBM protects against PI-induced excitotoxicity in the hippocampus in vivo

**Fig. 4:**
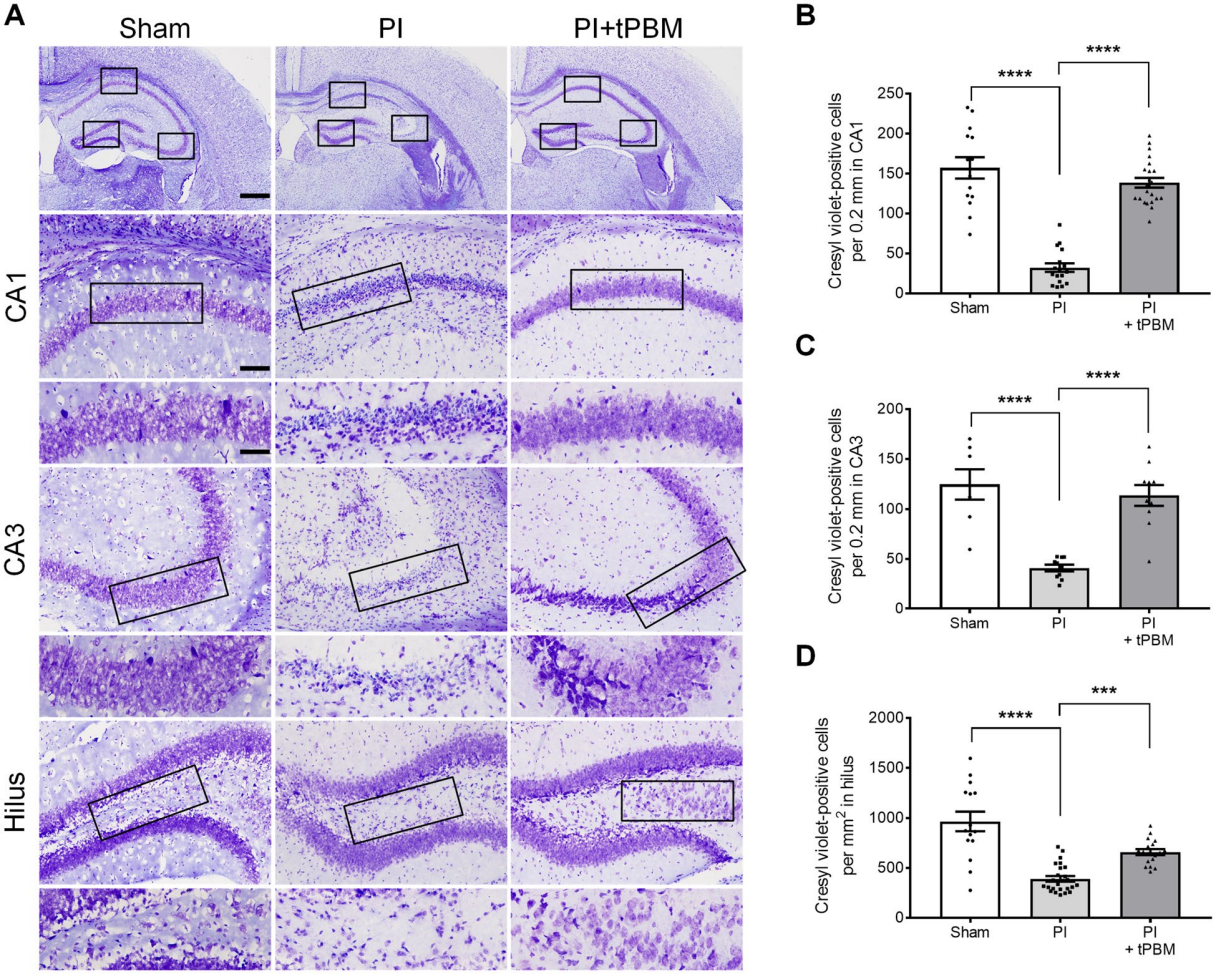
Neuroprotective effect of tPBM in the mouse hippocampus after PI-induced excitotoxicity as evaluated by CV staining. **A** Representative images of CV-stained brain coronal sections at 7 days after PI-induced SE. Treatment with 825 nm tPBM protected the CA1, CA3, and hilus cells of the hippocampus at 4 h after PI injection. Insets are zooms of the boxed areas showing CA1, CA3, and hilus hippocampal regions. Higher magnification views of each hippocampal region enclosed by black boxes. The scale bars indicate 500, 100, and 50 μm, respectively. **B**–**D** Quantitative analyses of the neuronal damage in hippocampal CA1, CA3, and hilus in the PI and PI + tPBM groups. CA1, cornu ammonis1; CA3, cornu ammonis 3. Data are expressed as the mean ± SEM; ***p < 0.001, ****p < 0.0001 relative to PI (ANOVA with the Bonferroni test)

To assess the effects of tPBM on an in vivo model of excitotoxicity, we adopted the well-established PI-induced epileptic excitotoxicity model that leads to extensive neuronal cell death in the hippocampus [45, 46]. Previous studies have shown that a large number of granule cells and pyramidal cells in CA1, CA3, and the hilus are associated with hippocampal formation, and their neuronal death by PI leads to severe brain damage [47–49]. We investigated whether tPBM protects against PI-induced neuronal excitotoxicity including apoptosis. tPBM with 825 nm laser was used to irradiate the depilated mouse head at 6 Joules (10 mW/cm^2^, 10 min) at 4 h after PI injection. Neuronal death was confirmed by performing cresyl violet (CV) and Fluoro-Jade B (FJB) staining after PI intake. The CV-stained sections of the hippocampus from the sham group on day 7 showed an intact layering pattern in CA1 (157.21 ± 13.37 cells/0.2 mm, n = 14), CA3 (124.36 ± 15.19 cells/0.2 mm, n = 7) and the hilus (965.76 ± 97.57 cells/mm^2^, n = 15). Compared to the sham group, CV-positive cells were markedly reduced in CA1 at 40.94 ± 10.02 cells/0.2 mm (n = 18), followed by CA3 at 36.89 ± 3.23 cells/0.2 mm (n = 10) and the hilus at 391.78 ± 27.58 cells/mm^2^ (n = 25) in the PI group (Fig. 4B–D). We also observed many pyknotic nuclei in CA1, CA3, and the hilus of the PI group. At 4 h after PI intake, tPBM was performed with 825 nm transcranially. The penetration rate of laser application was about 63.6% (Fig. 1C). In the tPBM group, CV-positive cells were significantly increased in CA1 at 138.48 ± 6.11 cells/0.2 mm (n = 22), CA3 at 113.50 ± 10.29 cells/0.2 mm (n = 10), and the hilus at 658.40 ± 29.74 cells/mm^2^ (n = 18) compared to the PI group (Fig. 4B–D). Similar results were obtained after 3 days of PI (Additional file 1: Fig. S2). These results suggest the tPBM upregulated the neuronal population, which was altered by PI application. We have failed to observe the morphologic changes or decreased neuronal population in other brain area (Additional file 1: Fig. S3).

**Fig. 5:**
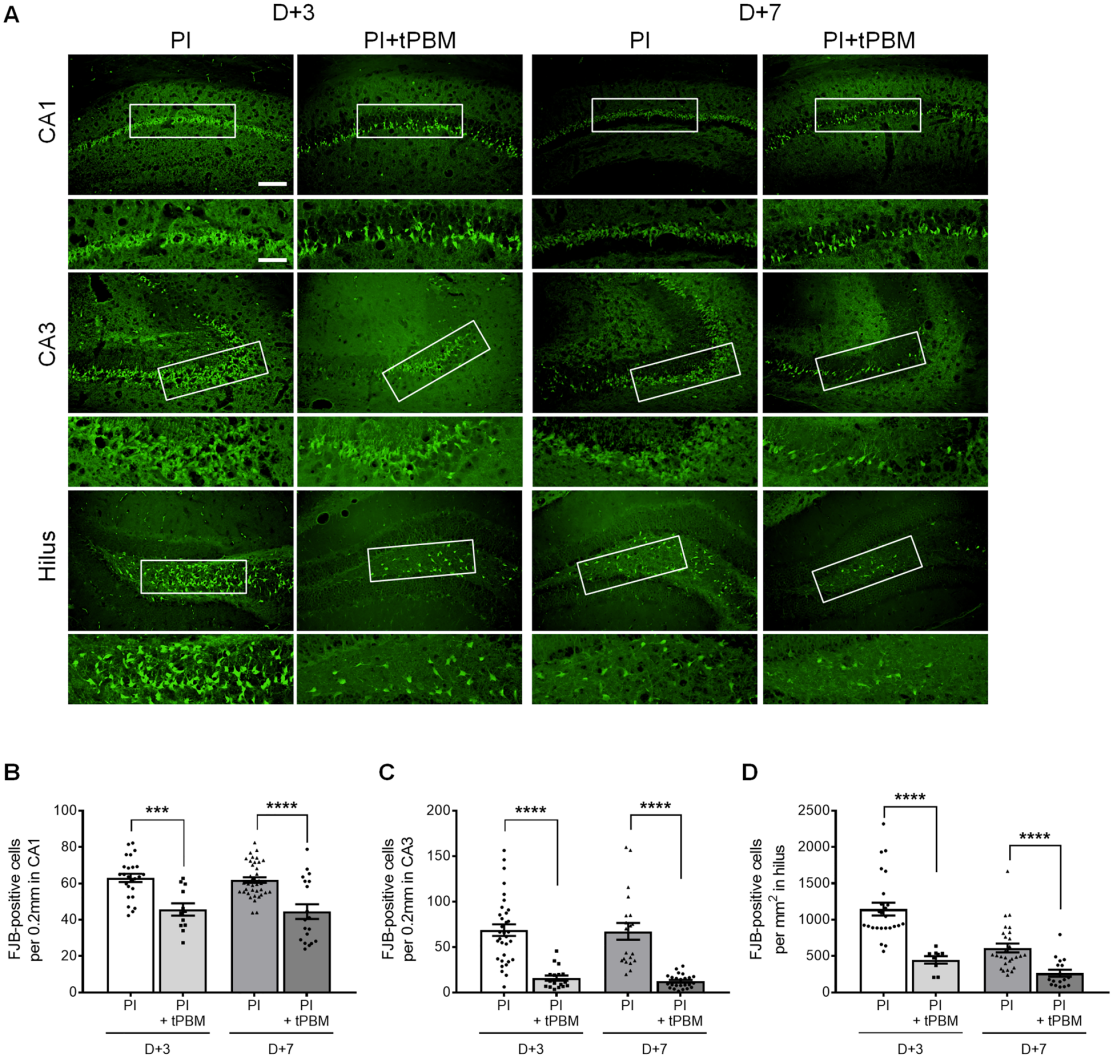
tPBM reduced neuronal apoptosis in the mouse hippocampus after PI-induced excitotoxicity. **A** Representative images of FJB-stained brain coronal sections at 3 and 7 days after PI-induced SE. Treatment with 825 nm tPBM protected the CA1, CA3 and hilus cells of the hippocampus at 4 h after PI injection. Higher magnification views of each hippocampal region enclosed by the black boxes. The scale bars indicate 100 and 50 μm. **B**–**D** Quantitative analyses of the apoptotic neurons in hippocampal CA1, CA3, and hilus in the PI and PI + tPBM groups. CA1, cornu ammonis1; CA3, cornu ammonis 3. Data are expressed as the mean ± SEM; ***p < 0.001, ****p < 0.0001 relative to each PI group (Student’s *t*-test)

In parallel with the CV staining data, we confirmed the clear increase in degenerating neurons stained with FJB after 3 and 7 days of PI intake in the hippocampal subfields (3 days: CA1, 63.1 ± 2.2, n = 25; CA3, 68.7 ± 6.4, n = 33; hilus, 1146.7 ± 88.5, n = 25 and 7 days: CA1, 61.9 ± 1.6, n = 38; CA3, 67.3 ± 9.3, n = 20; hilus, 611.6 ± 60.8, n = 27) (Fig. 5). Furthermore, neuronal apoptosis was significantly decreased in all subfields of the hippocampus in the tPBM-treated mice after 3 and 7 days of PI (3 days: CA1, 45.7 ± 3.3, n = 12; CA3, 15.9 ± 2.9, n = 17; hilus, 448.0 ± 51.0, n = 9 and 7 days: CA1, 44.5 ± 4.0, n = 19; CA3, 12.3 ± 1.5, n = 24; hilus, 264.5 ± 47.6, n = 17). However, FJB-positive cells were not detected in the hippocampal regions of the sham group (data not shown). Quantitative analyses of CV-stained cells and FJB-positive cells showed that tPBM treatment induced robust effects against PI-induced neuronal excitotoxicity in the hippocampus of an in vivo animal excitotoxicity model.

### tPBM decreases neuroinflammation caused by PI-induced neurotoxicity in the hippocampus in vivo

**Fig. 6:**
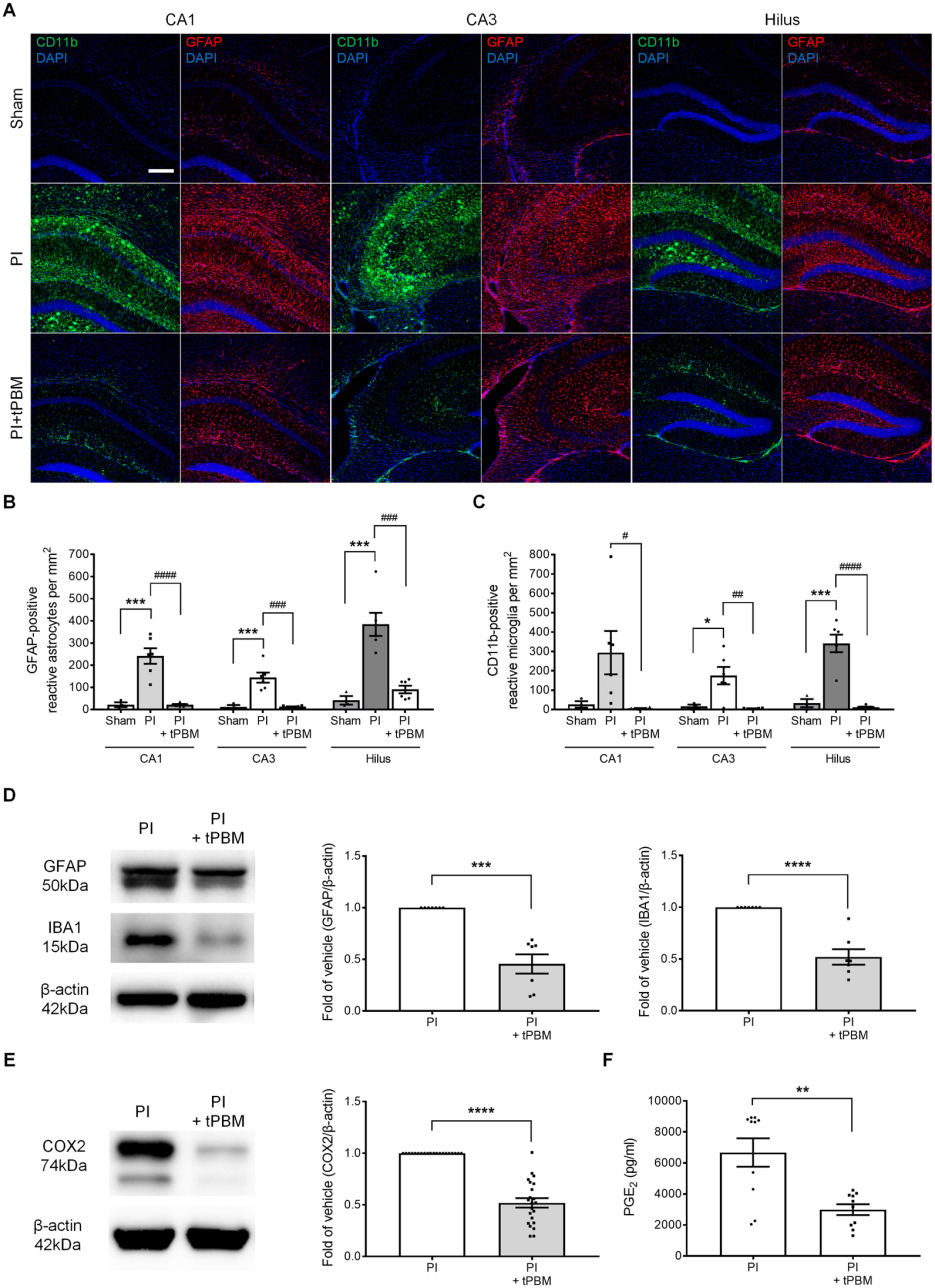
tPBM diminished PI-induced neuroinflammation in the mouse hippocampus. **A** Activation of microglia and astrocyte in CA1, CA3 and hilus was confirmed 7 days after PBM in PI-induced mouse excitotoxicity. IHC-stained CD11b and GFAP show the reactive form of microglia and astrocytes in each hippocampus region. Conditions were untreated sham, PI only and PI + tPBM with 825 nm groups. The stained images show that the number of reactive astrocytes and microglia was significantly lower in the 825 nm treated group compared to the PI only group. The scale bars indicate 100 μm. **B**, **C** The numbers of GFAP-immunostained reactive astrocytes and CD11b-immunostained reactive microglia in hippocampal CA1, CA3, and hilus were analyzed. These analyses revealed that tPBM significantly reduced the number of reactive glial cells in the hippocampus. Data are expressed as the mean ± SEM; *p < 0.05, ***p < 0.001 relative to sham, ^#^p < 0.05, ^##^p < 0.01, ^###^p < 0.001, ^####^p < 0.0001 relative to PI (ANOVA with the Bonferroni test). **D**, **E** GFAP, IBA1 and COX2 protein levels of homogenized hippocampus tissues were determined by western blotting. β-actin was used as a loading control. Data analysis was performed using ImageJ software by measuring the integrated band densities following background subtraction. Full-length blots/gels are presented in Additional file 1: Fig. S3. Data are expressed as the mean ± SEM; ***p < 0.001 relative to PI (Mann–Whitney *U* test), ****p < 0.0001 relative to PI (Student’s *t*-test). **F** Quantitative analyses of the prostaglandin E_2_ in homogenized hippocampus tissues in the PI and PI + tPBM groups. Data are expressed as the mean ± SEM; **p < 0.01 relative to PI (Student’s *t*-test)

Extensive astrocyte and microglia activation (gliosis) is associated with neuroinflammation caused by PI-induced neurotoxicity [50] and gliosis can lead to secondary damage by the immune responses [51, 52]. To establish whether tPBM attenuates PI-induced astrocyte and microglia activation, IHC analysis using astrocyte and microglia markers was performed on day 7 after treatment with PI (glial fibrillary acidic protein (GFAP) and cluster of differentiation molecule 11B (CD11b), respectively). GFAP and CD11b were not expressed in the CA1, CA3, and hilus in the sham group. However, GFAP and CD11b expression was significantly increased in all hippocampal regions in the PI group (Fig. 6A). Quantitative analyses of the number of GFAP- and CD11b-positive cells showed that GFAP-positive cells increased after 7 days of PI treatment (CA1, 241.1 ± 35.3, n = 6; CA3, 143.8 ± 22.8, n = 6; hilus, 384.3 ± 52.0, n = 6; Fig. 6B), and CD11b-positive cells increased after 7 days of PI treatment (CA1, 294.0 ± 111.8, n = 6; CA3, 175.2 ± 44.9, n = 6; hilus, 341.6 ± 45.6, n = 6; Fig. 6C). Next, quantitative analyses of the number of reactive astrocytes and microglia were conducted after tPBM treatment. The number of reactive astrocytes and microglia was significantly decreased in the hippocampal subfields compared with the PI group (GFAP: CA1, 21.1 ± 3.5, n = 6; CA3, 12.5 ± 1.7, n = 6; hilus, 90.2 ± 17.3, n = 6; CD11b: CA1, 5.9 ± 1.7, n = 6; CA3, 5.4 ± 1.1, n = 6; hilus, 11.6 ± 3.5, n = 6; Fig. 6B, C). Western blot analysis confirmed that reactive astrocytes and microglia were significantly decreased in the PI + tPBM group (GFAP, 0.45 ± 0.09, n = 7; ionized calcium binding adaptor molecule 1 (Iba1), 0.52 ± 0.07, n = 7; Fig. 6D, Additional file 1: Fig. S4). This reactive gliosis triggered the inflammatory process including chemokines, cytokines, prostaglandins, and extracellular proteases. Figure 6E, F and Additional file 1: Fig. S5 show that tPBM reduced neuroinflammatory mediators such as cyclooxygenase-2 (COX2), prostaglandin E_2_ (PGE_2_), interleukin 1beta (IL-1β), interleukin-6 (IL-6) and tumor necrosis factor alpha (TNF-α). Taken together, these data demonstrated that tPBM reduced reactive gliosis-induced neuroinflammation, which triggered the inhibition of secondary neuronal necrosis on day 7 after PI intake.

### Investigation of genes upregulated by tPBM and confirmation of tPBM protection against PI-induced synaptic degeneration in the hippocampus in vivo

**Fig. 7:**
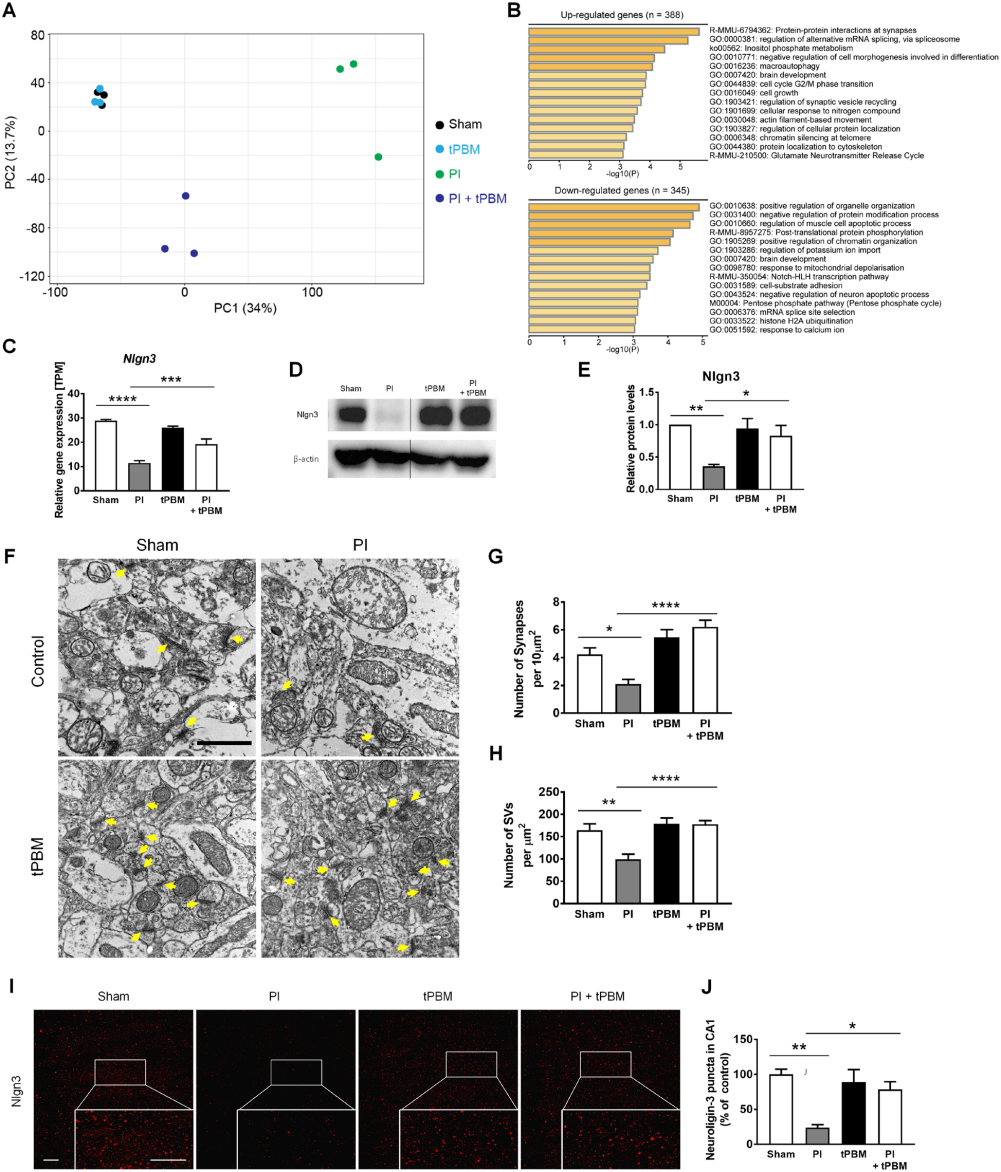
Investigation of genes upregulated by tPBM and confirmation of tPBM protection against PI-induced synaptic degeneration in the hippocampus in vivo. **A** Principal component analysis (PCA) of RNA-seq data. Sham (control), tPBM, PI, and PI + tPBM groups are plotted on the PCA plot according to their overall gene expression profiles. **B** Upregulated and downregulated biological pathways in the bar plots were identified using Metascape [37] with the DEGs. **C** Quantitative analysis of *Nlgn3* gene expression by TPM level. **D** Nlgn3 of the hippocampus in mouse was analyzed by western blotting with anti-Nlgn3 antibody. Full-length blots/gels are presented in Additional file 1: Fig. S6. **E** In the bar graph, quantification of the normalized band intensities (ratio of Nlgn3 protein to β-actin) indicated that Nlgn3 significantly increased in the presence of tPBM. **F** Representative TEM images of synapses of the hippocampus after PI and tPBM. Yellow arrows indicate distinct synapses. The scale bars indicate 1 μm. **G**, **H** Bar graphs show that the number of synapses and synaptic vesicles (SVs) of hippocampus were significantly changed by PI and tPBM. Bar graph shows that number of synapses and SVs were significantly altered by PI and tPBM. **I**, **J** Representative immunofluorescent images and quantification for Nlgn3 (red) after PI and tPBM. The scale bars indicate 20 μm. Bar graph shows that puncta of neuroligin-3 in CA1 were significantly altered by PI and tPBM. All data are expressed as the mean ± SEM; *p < 0.05, **p < 0.01, ***p < 0.001, ****p < 0.0001 compared with PI (ANOVA with the Bonferroni test)

**Fig. 8:**
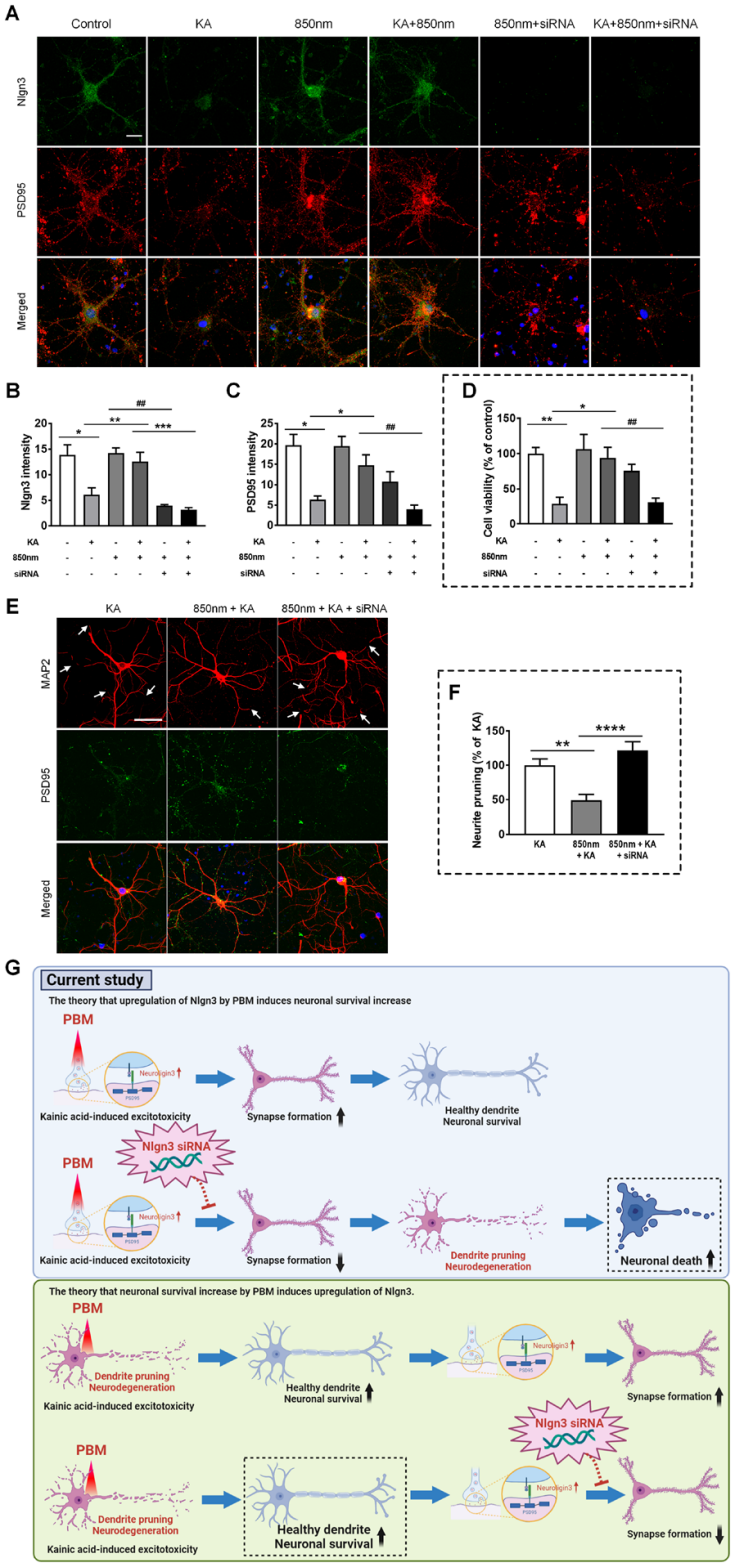
Nlgn3 gene silencing by Nlgn3 siRNA reduced synapses which are increased by PBM against the kainic acid-induced excitotoxicity, and led to neuronal cell death. **A** Representative immunofluorescent images for Nlgn3 (green) and PSD95 (red) after transfection and the treatment of kainic acid (KA) or 850 nm LED. Primary hippocampal neurons transfected with small interfering RNA (siRNA) targeting the Nlgn3 gene for 24 h. The scale bars indicate 20 μm. **B**, **C** Bar graphs show that the quantitative intensity analysis of neuroligin-3 and PSD95 after siRNA, kainic acid, and 850 nm LED. Data are expressed as the mean ± SEM; *p < 0.05, **p < 0.01 compared with KA, ***p < 0.001 compared with KA + 850 nm, ^##^p < 0.01 compared with 850 nm or KA + 850 nm (ANOVA with the Bonferroni test). **D** The bar graph shows that cell viability after siRNA, kainic acid, and 850 LED by DAPI counting. Data are expressed as the mean ± SEM; *p < 0.05, **p < 0.01 compared with KA, ^##^p < 0.01 compared with KA + 850 nm (ANOVA with the Bonferroni test). **E** MAP2 images showed the dendritic pruning by kainic acid (white arrow). The scale bars indicate 50 μm. **F** The bar graphs show that the neurite pruning was significantly higher in the Nlgn3 siRNA treated group compared to the 850 nm + KA group. Data are expressed as the mean ± SEM; **p < 0.01, ****p < 0.0001 relative to 850 nm + KA group (ANOVA with the Bonferroni test). **G** Schematic diagram of the current study indicated that Nlgn3 siRNA application with PBM and kainic acid leads to damage to neurons and dendrites after the synapse loss. If Nlgn3 is upregulated because PBM increases neuronal survival, then neuronal survival should also increase despite the application of Nlgn3 siRNA, but it did not

Next, we investigated whether tPBM regulates the genes related to biological pathways, such as synaptic modification, in the early phase of PI-induced excitotoxicity. For RNA-sequencing to reveal the possible mechanisms of tPBM in hippocampal neuronal excitotoxicity, animals were sacrificed at 1 h after tPBM application (5 h after PI). To identify genes affected by tPBM, changes in the gene expression levels were analyzed using RNA-sequencing. We observed minimal differences in overall gene expression profiles between control and tPBM only groups, suggesting that tPBM regulates only post-insult neuro recovery processes without interfering with normal physiology (Fig. 7A). When tPBM was introduced after inducing excitotoxicity with PI, the overall gene expression profile of the PI + tPBM group was clearly different from those of the other groups. This result clearly provides evidence that PBM has a distinct role in the post-insult neuro recovery process. Gene ontology analyses (between SE and tPBM with SE) revealed that several biological pathways related to the brain were significantly affected (Additional file 1: Fig. S6). Above all, the result showed protein–protein interactions at synapses as the key enriched gene ontology categories and Nlgn3 was identified in the two categories (Fig. 7B and Additional file 1: Fig. S6). Synaptic degeneration precedes neuronal apoptosis in several neurodegenerative processes by excitotoxicity [53–55]. Nlgn3 is closely related to synaptic formation [56–59]. Relative gene expressions (TPM) of Nlgn3 by RNA sequencing data among four groups (Sham: control; PI: SE model only; tPBM only; tPBM + PI) were compared. There were statistical difference of relative gene expression among groups. PI group showed statistically lower expression of Nlgn3 compared to Sham group (Sham, 28.91 ± 0.29, n = 3 vs. PI, 11.47 ± 0.55, n = 3). PI + tPBM group showed statistically higher expression of Nlgn3 compared to PI only group (PI + tPBM, 19.22 ± 1.25, n = 3; Fig. 7C). To confirm the finding of RNA sequencing about Nlgn3 in tPBM, we performed the western blot analysis and detected the expression of Nlgn3 using immunofluorescence staining. Western blot analysis confirmed that Nlgn3 were significantly decreased in the PI group compared to Sham and PI + tPBM group (Fig. 7D, E and Additional file 1: Fig. S7; PI, 0.36 ± 0.03, n = 11; PI + tPBM, 0.83 ± 0.16, n = 11). TEM images showed that synapses and synaptic vesicle (SV)s in hippocampus was significantly decreased in the PI group compared to Sham and PI + tPBM group (Fig. 7F–H; Synapses: Sham, 4.23 ± 0.48%, n = 9 vs. PI, 2.12 ± 0.33%, n = 8 vs. PI + tPBM, 6.22 ± 0.48%, n = 9; SVs: Sham, 151.08 ± 14.74, n = 12 vs. PI, 115.75 ± 15.09, n = 14 vs. PI + tPBM, 192.08 ± 11.97, n = 18). Immunofluorescence staining showed that Nlgn3 and Synaptophysin level in CA1 and DG of hippocampus was significantly decreased in the PI group compared to Sham and PI + tPBM group (Fig. 7I-J; Nlgn3: Sham, 100 ± 7.48%, n = 5 vs. PI, 23.88 ± 4.2%, n = 5 vs. PI + tPBM, 78.51 ± 11.03%, n = 5; Additional file 1: Fig. S8; Synaptophysin: Sham, 100 ± 7.8%, n = 4 vs. PI, 11.21 ± 1.13%, n = 4 vs. PI + tPBM, 51.12 ± 5.88%, n = 4). For the evaluation of inhibitory synapses, densities of Gephyrin puncta in CA1 were analyzed. There were no statistical difference of Gephyrin puncta density among groups (Sham, PI, tPBM and PI + tPBM; Additional file 1: Fig. S9). This result indirectly suggests that damaged or protected synaptic puncta would be mostly from excitatory synapse rather than inhibitory. These results indicate that tPBM can serve as an important regulator of synapses against PI-induced synaptic loss in the hippocampus. Again, to confirm the finding of RNA sequencing, western blot and epifluorescence analysis about Nlgn3 in tPBM, we detected the expression of Nlgn3, PDS 95 and MAP2 (Neurite) using immunofluorescence staining in vitro (4 h) using siRNA to inhibit the Nlgn3 gene expression in vitro. Immunofluorescence staining in vitro showed that Nlgn3 and PSD 95 level in primary hippocampal culture was increased in sham and tPBM + kaininc acid groups compared to kainic acids only groups (Fig. 8A). Meanwhile, in the group of tPBM and siRNA that were treated together with kainic acid, we confirmed the decrease Nlgn3 and PDS95 expression (Fig. 8A–C). Our results showed that Nlgn3 siRNA reduced the Nlgn3 (3.11 ± 0.48%, n = 7) and PSD95 intensity (3.94 ± 1.1%, n = 7) compared with 850 nm + kainic acid group by 12.54 ± 1.85% (n = 10), and 14.79 ± 2.54% (n = 12), respectively (Fig. 8B, C). Additionally, there was statistically significant decrease of Nlgn3 in siRNA + PBM compared to PBM (PBM, 14.22 ± 1.0, n = 3 vs. siRNA + PBM, 3.97 ± 0.22, n = 11; Fig. 8B). But, there were no significant decrease of PSD 95 in siRNA + PBM compared to PBM (PBM, 19.49 ± 2.36, n = 4 vs. siRNA + PBM, 10.75 ± 2.45, n = 8; Fig. 8C). This outcome correlates to the results of RNA sequencing and other in vivo outcomes. We also observed that Nlgn3 siRNA treated group not block neurite pruning (121.69 ± 12.58%, n = 11) compared to the 850 nm + kainic acid group (49.06 ± 8.64%, n = 15, Fig. 8F), indicating reduced neuritis pruning by PBM was reversed as well by silencing of Nlgn3 by siRNA. In the assessment of cell viability (24 h), as already demonstrated in earlier in vitro study kainic acid group showed decreased cell viability compared to control and PBM + kainic acid group. PBM + kainic acid + siRNA group showed decreased cell viability compared to PBM + kainic acid group (KA + PBM, 93.98 ± 15.06%, n = 6 vs. KA + PBM + siRNA, 31.17 ± 6.04%, n = 8; Fig. 8D). Ciliary neurotrophic factor receptor, cytoplasmic polyadenylation element-binding protein 4, C-X3-C motif chemokine ligand 1, and syntaxin-binding protein 1 were among the genes that were downregulated (Additional file 1: Fig. S6). These genes are related to the negative regulation of the neuron apoptotic process pathway, which is closely related to neuronal apoptosis [60–63]. Overall, these results showed that treatment with tPBM contributed to the gene regulatory networks of neuronal functions in the mouse brain including Nlgn3.

### tPBM attenuates PI-induced acute seizure and behavioral abnormalities in vivo

**Fig. 9:**
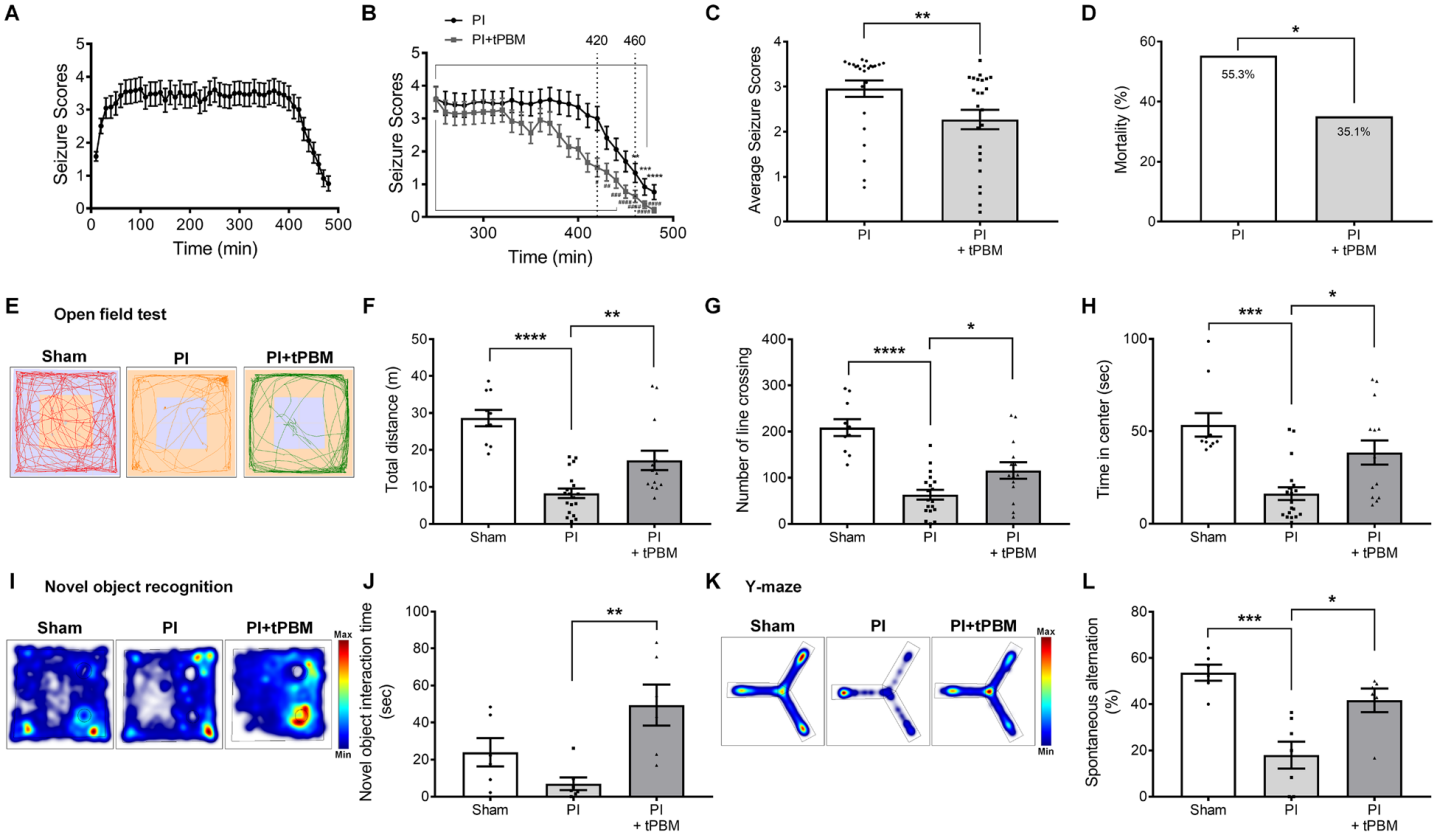
tPBM attenuated PI-induced seizures and behavioral dysfunctions. **A** The time course of PI-induced seizures in C57BL/6 mouse after administration of PI (320 mg/kg, i.p.). Average seizure scores were plotted based on the Racine scale. **B** Note significantly reduced seizure scores by tPBM. Data are expressed as the mean ± SEM; **p < 0.01, ***p < 0.001, ****p < 0.0001 relative to 250 min PI, ^#^p < 0.05, ^##^p < 0.01, ^###^p < 0.001, ^####^p < 0.0001 relative to 250 min PI + tPBM (Kruskal–Wallis with Dunn’s multiple comparisons test). **C** Average seizure scores were lower by tPBM. Data are expressed as the mean ± SEM; **p = 0.0015 relative to PI only groups (Student’s *t*-test). **D** Mortality following PI injection was reduced by tPBM. Data are expressed as the mean ± SEM; *p = 0.08 relative to PI (chi-square test). **E** Exploratory behavior in the open field test (OFT) was evaluated 7 days after tPBM in the PI-induced excitotoxicity mouse model. Representative traces of mice pattern activity (red, orange, or green line) resulting from EthovisionXT 12 software video tracking are depicted. **F**–**H** Bar graphs show that total distance, the number of line crossings and time spent in the center were significantly altered by tPBM. **I** Heatmap of the novel object recognition using EthovisionXT 12 software was altered by tPBM. **J** The bar graph shows that novel object interaction time was significantly increased by tPBM. **K** Heatmap of the Y-maze showed that spontaneous alternation was impaired by tPBM. **L** The bar graph shows that the percent of spontaneous alternation in the Y-maze was significantly above chance level by tPBM. Data are expressed as the mean ± SEM; *p < 0.05, **p < 0.01, ***p < 0.001, ****p < 0.0001 compared with PI (ANOVA with the Bonferroni test)

To determine whether these neuroregulatory effects of tPBM can alter PI-induced acute seizures, mice were treated with PI and the resulting seizures were videotaped and scored using the Racine scale every 10 min for 8 h. The seizure score increased progressively to 100 min, was maintained until 300 min, and then began to decrease (Fig. 9A). In the group treated with tPBM 4 h after PI injection, the seizure score significantly decreased rapidly (420 min, 3.00 at n = 63 vs. 1.52 at n = 62; p = 0.0363 (Fig. 9B). The average seizure scores during the period between 240 and 480 min after treatment with tPBM were significantly reduced by 2.27 ± 0.1 (n = 37) compared with the PI group (2.96 ± 0.05, n = 38; Fig. 9C). Consistent with the reduced seizure score, mortality over 7 days after PI injection was also reduced in tPBM-treated mice but without statistical significance (PI: 21/38, 55.3%; PI + tPBM: 13/37, 35.1%) (Fig. 9D). Collectively, our data indicate that tPBM significantly contributed to the recovery of PI-induced acute seizure in vivo.

PI-induced acute seizure and neurotoxicity are associated with behavioral abnormalities such as anxiety as well as locomotor and explorative hypoactivities in animal models [64–67]. Next, the tPBM regulation of PI-induced dysfunction of locomotor and explorative hypoactivities and anxiety-related behavior were investigated using the open-field test (OFT). Statistical analyses of total distance and number of line crossings revealed that tPBM-treated mice had increased locomotor and explorative hypoactivities compared with PI only-treated mice (Fig. 9E–G, Additional file 1: Fig. S10). During the OFT, time in the center zone was measured as anxiety-related behavior. tPBM-treated mice spent a significantly longer time in the center compared with PI only-treated mice (Fig. 9H). These results indicate that tPBM-treated mice had improved anxiety without PI-induced motor defects. Next, the novel object recognition test was used to examine whether the PI-induced acute seizure and treatment with tPBM affect recognition memory. Notably, tPBM-treated mice showed significantly higher preference for the novel object than did PI only-treated mice, which was greater than normal mice who tended to prefer the familiar object (Fig. 9I, J). To examine the effects of tPBM on hippocampal-dependent learning and memory, a less stressful and ethologically more relevant spatial-memory test, the Y maze test, was also used in this study. The sham mice entered more frequently into novel arms of the maze that were previously unvisited (Fig. 9K, L). In contrast, PI-treated mice showed no preference for the novel arm and entered randomly into different arms. Notably, tPBM-treated mice also showed significantly higher spontaneous alternation than the PI only group (Fig. 9K, L). These data indicate that holding spatial information, which depends on hippocampal working memory, was improved in tPBM-treated mice. Overall, these results clearly showed that tPBM improved PI-induced acute seizures and behavioral abnormalities such as cognitive dysfunction, anxiety, and explorative hypoactivity in mice.

## Discussion

Here, we used kainic acid-induced excitotoxicity as an in vitro epileptic model to mimic clinical seizure induced excitotoxicity. We studied the effects of PBM on neuronal function in rat hippocampal primary cultured neurons. Synaptic loss is an early step related to the damage of neuronal function in many neurodegenerative diseases. In this study, we used a quantitative synapse approach to monitor changes in synaptic connections during excitotoxicity-induced synaptic degeneration. The dendritic spine is a basic functional unit of the integration of neuronal circuits and a site of structural and functional synaptic plasticity. Spines are subject to early and selective damage during excitotoxicity. Almost all dendritic spines are contacted by excitatory synapses, rendering these postsynaptic structures vulnerable to conditions of excitotoxicity [68]. Excitotoxic alterations in synaptic proteins may contribute to the rapid disruption of neurological function occurring within minutes of energy depletion in the brain. Therefore, we also investigated dendritic morphological changes using neurite outgrowth analysis. We found that loss of PSD95 puncta and morphological degradation occurred within 4 h after treatment with kainic acid. This neuronal damage was progressive and finally triggered neuronal death, whereas, 850 nm LED-treated hippocampal neurons showed increased PSD levels and decreased dendritic damage. These results indicate that near infra-red PBM has potential as a neuroprotective application against kainic acid-induced excitotoxic neurodegeneration and related neuronal diseases.

We focused our study on epileptic seizures, particularly SE, since it is not only accompanied by a large increase of glutamate in brain fluids but there is also a tight correlation between PI-related brain damage and the development of chronic epilepsy [46, 69–73]. We assessed neurodegeneration and apoptosis using PI-induced SE as an epilepsy mouse model which is one of the most commonly studied chemical-inductive models for epilepsy [69, 71, 74–76]. Morphological analyses of the brain after PI-induced SE have demonstrated that the hippocampal subfield CA1 and hilus of the dentate gyrus are particularly susceptible to neuronal cell loss [75, 76]. In this study, compared with the sham controls, treatment with PI decreased the number of neurons in the hippocampal stratum pyramidale and the molecular layers of the CA1, CA3, and hilus 3 on day 7. Near infra-red tPBM was found to increase the number of neurons in the hippocampus after PI-induced excitotoxicity as shown by CV staining, and in line with the FJB staining data. We also investigated and found out that the synapto-protective effect of near infra-red PBM shown in an in vitro excitotoxicity model was reproduced in a PI-induced excitotoxicity mouse model.

We observed that there are minimal gene expression differences between control and PBM only group in RNA sequencing, suggesting that PBM is not interfering with normal physiology and modulating only the neuro-recovery process. As included in Fig. 7A, it is clearly noted that after PBM and PI gene expression pattern is distinctively different compared to others. This outcome clearly proves the distinct role in PBM during after PI insult. From our results, it was confirmed that PBM blocked the Nlgn3 gene decrease induced by PI in the pathologic condition of SE, and this was supported by significant increase of the protein level of Neuroligin-3 by western blotting. It has been reported that Nlgn3 expression correlates with synaptic connections in the hippocampus [77]. Lack of upregulation of Nlgn3 by PBM in no insult status would be due to maturation of synapses and neuronal structures. This result corresponds to publication showing no metabolic change by PBM in healthy animal brain. The authors of this manuscript claim that PBM therapy would be effective in subjects whose normal physiology is compromised [78]. In addition, no response of PBM in other brain areas where minimal damage from PI induced SE is expected (Additional file 1: Fig. S3) supports the theory as well. It is possible that PBM is altering the neuronal survival itself and more surviving neurons are responsible for the difference of Nlgn3 expression. But the time point of RNA sequencing is 1 h after tPBM and that of observation of degeneration of hippocampal neurons was more than 72 h later. We believe that with the fact that RNA sequencing is performed at very early stage and the fact that PBM altered the synaptic degeneration by kainic acid at early time point without major changes in dendrites (Fig. 3A–D), it is highly possible that this Nlgn3 gene expression alteration is resulted earlier than time point of neuronal population alteration. In fact, in the dendritic morphologies and cell viability with PBM, kainic acid and siRNA, we have observed degeneration of dendrites and decreased cell viability compared to PBM and kainic acid (Fig. 8D, F). If the PBM was to recover cellular health against kainic acid and thereby Nlgn3 expression was intact to form synaptic puncta (secondarily), siRNA application with kainic acid and PBM would result in only reduction of PSD95 puncta without damage to neuron and other cellular structure, which is opposite to our current observation (Fig. 8G).

The decreases of neuro-inflammation could be sequential or collateral outcome to excitotoxicity [79, 80]. A strong relationship between neuroinflammation and epileptic seizures has been reported [81, 82]. In both animal models and patients with epilepsy, increasing evidence has shown that elevated inflammation in the brain during epileptic seizures plays a role in persistent seizures and long-term sequelae. Our results showed that near infra-red tPBM reduced neuroinflammation through the decrease of reactive gliosis possibly due to smaller primary neuronal damages. We also showed that the near infra-red tPBM inhibited production of COX2, PGE_2_, and pro-inflammatory cytokines as well as gliosis. The significant reduction of seizure score in tPBM animal at later stage could be related to these findings.

We performed RNA-sequencing to evaluate tPBM-induced transcriptional changes in the hippocampus of an excitotoxicity-induced mouse model or near infra-red tPBM-treated mouse groups. Our results showed that various genes related to neuronal development processes were upregulated in the tPBM-treated groups. In particular, genes related to synaptic formations were markedly increased including Nlgn3, leucine rich repeat transmembrane neuronal 4 (*Lrrtm4*), and *Dlgap1*. These upregulated genes indicate intracellular cell adhension molecules, proteins neuroligin, and PSD-95, respectively. Appropriate synaptic formation is essential for neuronal function and depends on the intercellular protein–protein interactions of cell adhesion molecules at synaptic clefts. Among them, specifically, Nlgn3 showed robust increase after PBM and silencing the Nlgn3 reversed the positive effect of PBM in in vitro. Cell adhension molecules play critical roles in determining synapse specificity by mediating the initial target recognition between pre- and post-synaptic neurons during synapse formation [83, 84]. Synaptic components exist in abundance at pre- and post-synaptic terminals in the early stages of synapse development with the support of cell adhesion molecules interactions [85, 86]. During the later stages of synapse development and in mature synapses, cell adhesion molecules regulate synaptic structure and function.

Inevitably, epileptic seizure can lead to cognitive dysfunction, mental behavioral abnormalities, and decreased quality of life in patients with epilepsy. As shown in our behavior test, the near infra-red tPBM treatment accelerated seizure termination latency and decreased the average seizure scores as well as mouse mortality. The locomotor and explorative hypoactivities in experimental rodent models of epilepsy, such as SE, have been previously observed from monitoring behavioral abnormalities in behavioral tests such as the OFT [87–89]. Notably, the tPBM-treated group showed increased locomotor and explorative activities and decreased anxiety in the OFT. In addition, tPBM also enhanced cognitive function via novel object recognition and the Y maze test. Thus, an association between the behavioral impairment and synapse and neuronal damage in hippocampus during epilepsy was confirmed as well as the protective effect of tPBM against epilepsy. This, functional improvement of animals, raises the possibility of clinical uses of tPBM for both acute stage to reduce seizure and later stage to reduce complication of cognitive dysfunction.

Clinically, only pharmacologic treatment, which harbors potential complications, is the current option for patients with an acute attack of epilepsy. Nevertheless, substantial portion of epileptic patients do not respond to these medications. Due to the non-invasive characteristics of tPBM, if validated effective for neuroprotection against excitotoxicity, PBM could provide a wide spectrum of benefits to patients who suffer from epilepsy and complications associated with the disease. However, due to anatomical differences between species, such as a thicker skull and deeper location of structures associated with the current study, optimization of PBM for clinical application is required. The possibility of development of port of delivery and surgical methods for delivery port exists. Because increase of the PBM power in current wavelength (which has a relatively fair penetration rate) is available, application to children who have relatively thin skull thickness could be possible. Conversely, for PBM application to adults, various methods, such as multiway methods that use multiple light delivery devices simultaneously, in different ways, to reach the required amount of energy, could be utilized.

## Conclusions

Here, we provide evidence that PBM is contributed to neuronal functions between hippocampal neurons in TLE pathological processes. PBM plays a critical role in excitotoxicity by regulating neuronal structure, synapses and survival. In addition, we found that PBM attenuated hippocampal neuroinflammation by modulating the reactive gliosis. Moreover, using RNA sequencing, we demonstrate for first time that PBM stimulates synaptic formation and anti-apoptotic pathway resulting in regulatory networks of neuronal functions. Eventually, these neuroprotective effect of PBM improves acute seizure in initial phase of status epilepticus and ameliorates behavioral cognitive dysfunctions such as hypoactivity, anxiety and working memory.

## Supplementary Information

**Additional file 1: Figure S1.** Full-length blots of synaptic markers. (A) β-actin of PSD95, (B) PSD95. **Figure S2.** CV staining on 3 days after PI-induced excitotoxicity (*p < 0.0001 relative to Sham; **p < 0.0001 relative to PI). **Figure S3.** CV staining of thalamus and cortex regions on 7 days after PI-induced excitotoxicity. **Figure S4.** Full-length blots of neuroinflammatory markers. (A) β-actin of GFAP and IBA1, (B) GFAP, (C) IBA1, (D) β-actin of COX2, (E) COX2. **Figure S5.** RT-PCR results of pro-inflammatory cytokines. **Figure S6.** Gene ontology analyses of differentially expressed genes in PI-induced excitotoxicity mouse samples compared to control samples. **Figure S7.** Full-length blots of neuroligin-3 antibody. (A1-3) Nlgn3, (B1-3) β-actin of Nlgn3. **Figure S8.** The evaluation of pre-synaptic terminals in the DG using anti-Synaptophysin. **Figure S9.** The evaluation of inhibitory synapses in the CA1 using anti-Gephyrin. **Figure S10.** The other OFT parameter analysis and Rotarod test results (*p < 0.05, **p < 0.01, ****p < 0.0001 relative to PI). **Videos S1–S6.** Six video files indicated each step about behavioral acute seizures based on a modified Racine scale.

## Data Availability

The datasets generated and/or analyzed during the current study are available from the corresponding author on reasonable request.

## Abbreviations

PBM: Photobiomodulation
TLE: Temporal lobe epilepsy
PI: Pilocarpine
SE: Status epilepticus
LLLT: Low-level laser therapy
NIR: Near-infrared
LED: Light-emitting diode
tPBM: Transcranial Photobiomodulation
CV: Cresyl violet
FJB: Fluoro-Jade B
PSD-95: Postsynaptic density protein 95
MAP2: Microtuble associated protein 2
GFAP: Glial fibrillary acidic protein
CD11b: Cluster of differentiation molecule 11B
COX2: Cyclooxygenase-2
PGE_2_: Prostaglandin E_2_
IL-1β: Interleukin 1beta
IL-6: Interleukin-6
TNF-α: Tumor necrosis factor alpha
IHC: Immunohistochemistry
Dlgap1: Disks large-associated protein 1
Camk2d: Calcium/calmodulin-dependent protein kinase II delta
Nlgn3: Neuroligin 3
Lrrtm4: Leucine rich repeat transmembrane neuronal 4
OFT: Open-field test
ROS: Reactive oxygen species
