# Supplementary material for: Photobiomodulation improves the synapses and cognitive function and ameliorates epileptic seizure by inhibiting downregulation of Nlgn3
Source: Cell Biosci. 2023 Jan 12;13:8. doi: 10.1186/s13578-022-00949-6 (PMC9837965; doi:10.1186/s13578-022-00949-6)
Supplement: Supplementary file 1 — Additional file 1: Figure S1. Full-length blots of synaptic markers. (A) β-actin of PSD95, (B) PSD95. Figure S2. CV staining on 3 days after PI-induced excitotoxicity (*p < 0.0001 relative to Sham; **p < 0.0001 relative to PI). Figure S3. CV staining of thalamus and cortex regions on 7 days after PI-induced excitotoxicity. Figure S4. Full-length blots of neuroinflammatory markers. (A) β-actin of GFAP and IBA1, (B) GFAP, (C) IBA1, (D) β-actin of COX2, (E) COX2. Figure S5. RT-PCR results of pro-inflammatory cytokines. Figure S6. Gene ontology analyses of differentially expressed genes in PI-induced excitotoxicity mouse samples compared to control samples. Figure S7. Full-length blots of neuroligin-3 antibody. (A1-3) Nlgn3, (B1-3) β-actin of Nlgn3. Figure S8. The evaluation of pre-synaptic terminals in the DG using anti-Synaptophysin. Figure S9. The evaluation of inhibitory synapses in the CA1 using anti-Gephyrin. Figure S10. The other OFT parameter analysis and Rotarod test results (*p < 0.05, **p < 0.01, ****p < 0.0001 relative to PI). Videos S1–S6. Six video files indicated each step about behavioral acute seizures based on a modified Racine scale. [file 13578_2022_949_MOESM1_ESM.docx]

**Additional file 1**

Figure S1: Full-length blots of synaptic markers. (A) β-actin of PSD95, (B) PSD95.

Figure S2: CV staining on 3 days after PI-induced excitotoxicity (*p < 0.0001 relative to Sham; **p < 0.0001 relative to PI).

Figure S3: CV staining of thalamus and cortex regions on 7 days after PI-induced excitotoxicity.

Figure S4: Full-length blots of neuroinflammatory markers. (A) β-actin of GFAP and IBA1, (B) GFAP, (C) IBA1, (D) β-actin of COX2, (E) COX2.

Figure S5: RT-PCR results of pro-inflammatory cytokines.

Figure S6: Gene ontology analyses of differentially expressed genes in PI-induced excitotoxicity mouse samples compared to control samples.

Figure S7: Full-length blots of neuroligin-3 antibody. (A1-3) Nlgn3, (B1-3) β-actin of Nlgn3.

Figure S8: The evaluation of pre-synaptic terminals in the DG using anti-Synaptophysin.

Figure S9: The evaluation of inhibitory synapses in the CA1 using anti-Gephyrin.

Figure S10: The other OFT parameter analysis and Rotarod test results (*p < 0.05, **p < 0.01, ****p < 0.0001 relative to PI).

Videos S1-S6: six video files indicated each step about behavioral acute seizures based on a modified Racine scale.

**Additional figures**

**
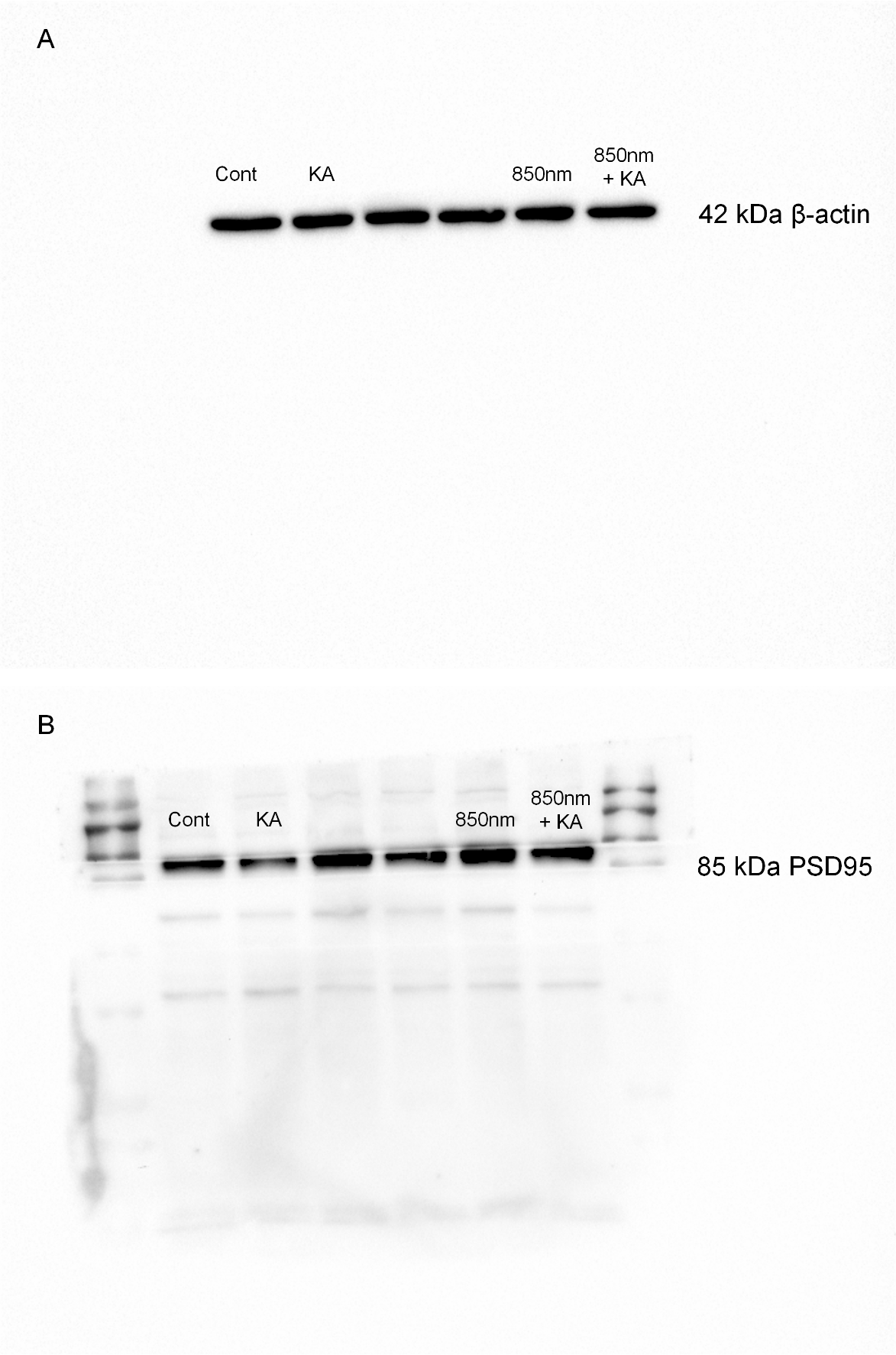
**

**Figure S1.** Full-length blots of synaptic markers. (A) β-actin of PSD95, (B) PSD95.


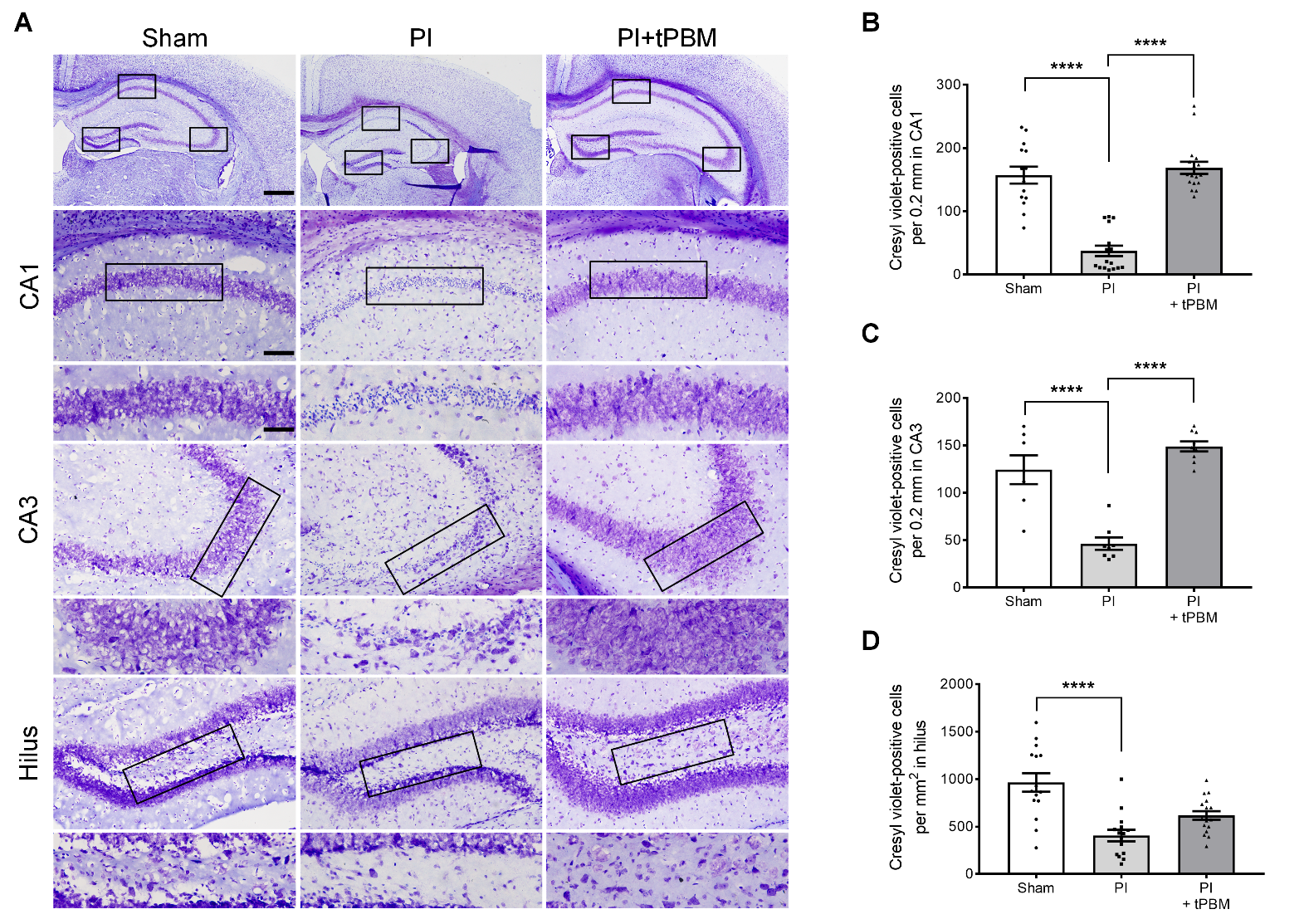


**Figure S2.** Neuroprotective effect of photobiomodulation in the mouse hippocampus 3 days after pilocarpine-induced excitotoxicity as evaluated by cresyl violet staining (****p < 0.0001 relative to PI). The scale bars indicate 500, 100, and 50 μm, respectively.


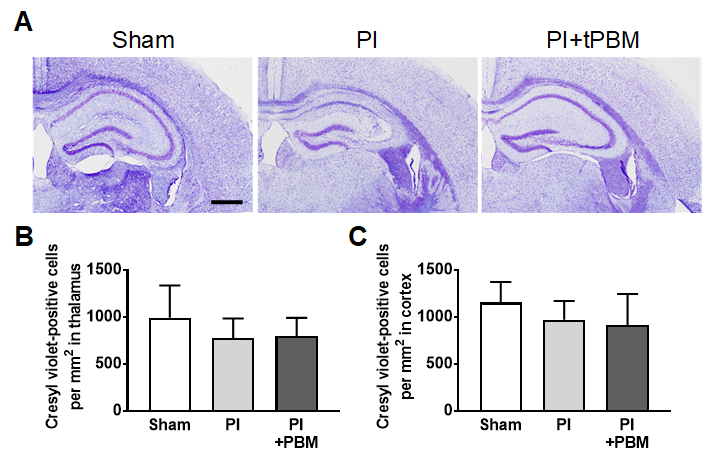


**Figure S3.** Photobiomodulation has no effect in the mouse thalamus and cortex regions 7 days after pilocarpine-induced excitotoxicity. Bar graphs show that there is no significant difference after the treatment of photobiomodulation (scale bar = 500 μm).

**
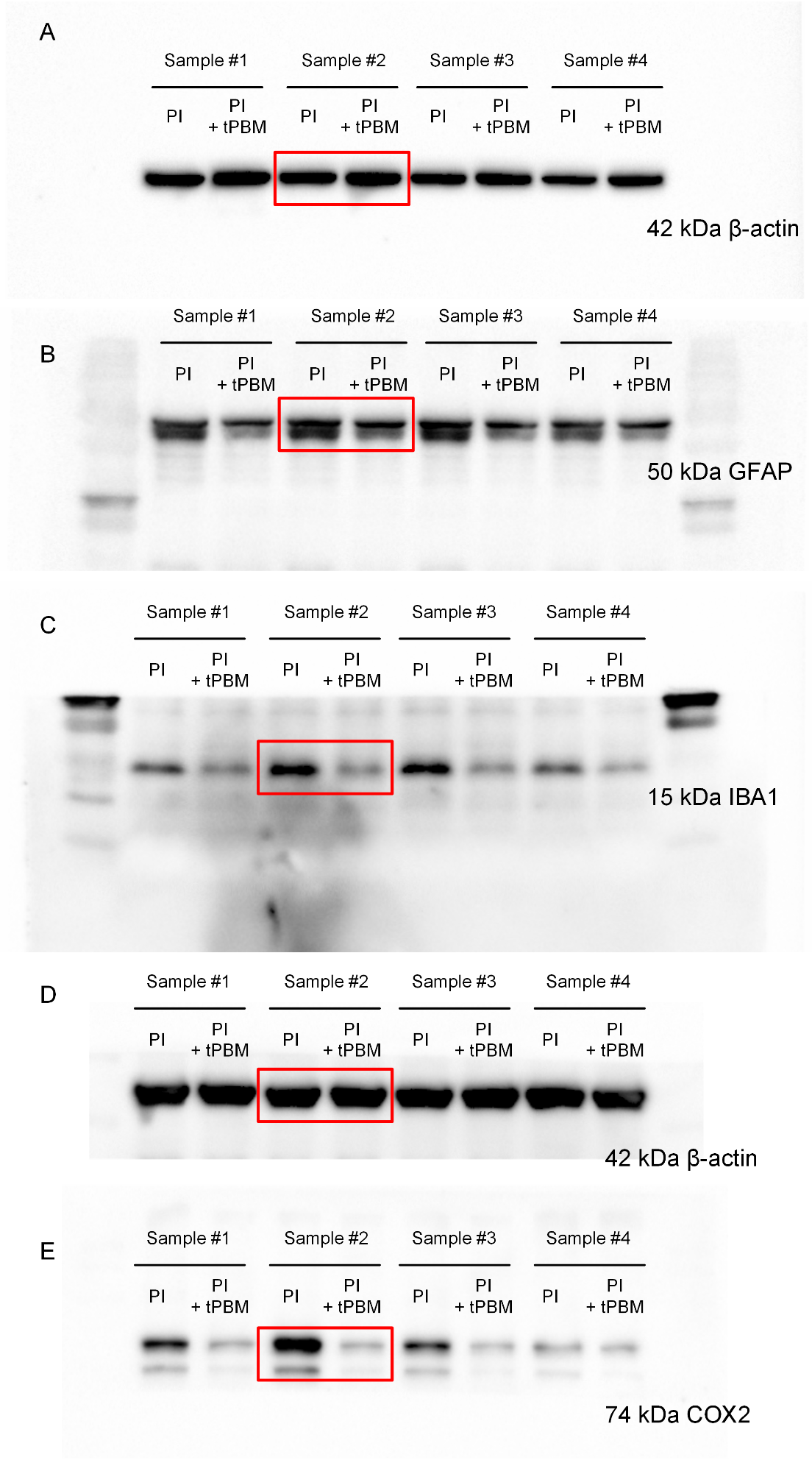
**

**Figure S4.** Full-length blots of neuroinflammatory markers. (A) β-actin of GFAP and IBA1, (B) GFAP, (C) IBA1, (D) β-actin of COX2, (E) COX2.


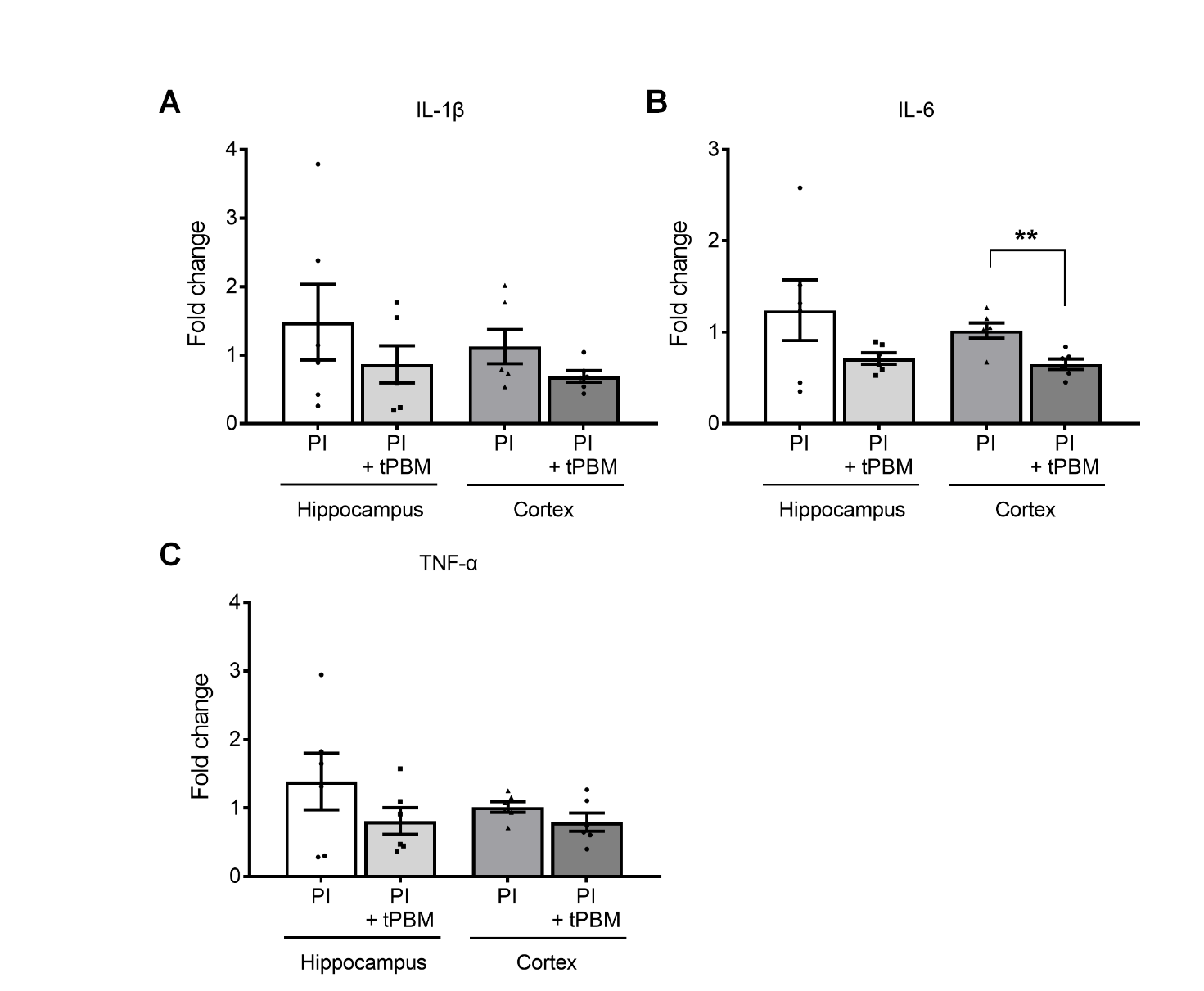


**Figure S5.** RT-PCR results of pro-inflammatory cytokines. (A) IL-1β (B) IL-6 (C) TNF-α


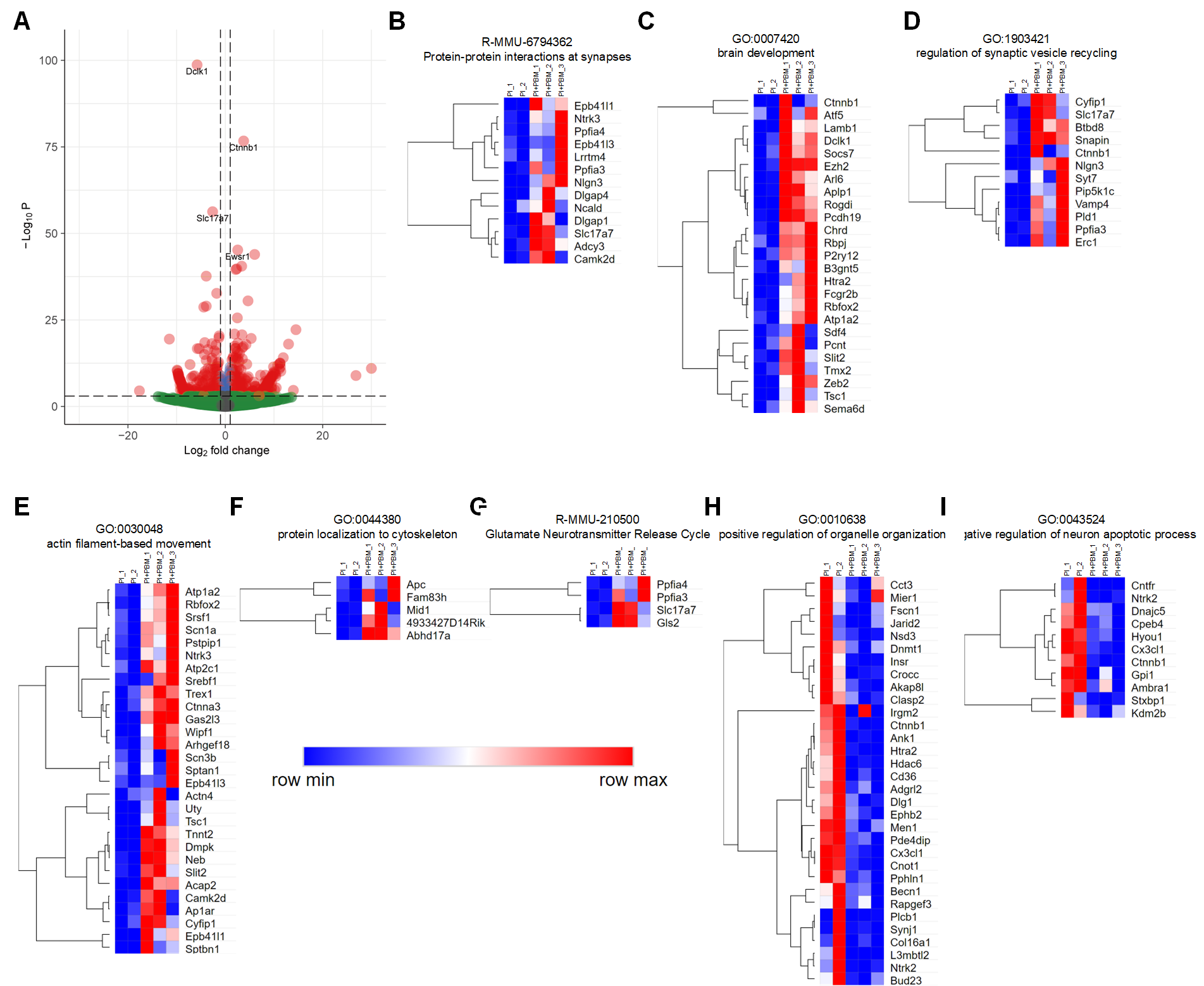


**Figure S6.** Gene ontology analyses of differentially expressed genes in PI-induced excitotoxicity mouse samples compared to control samples. (A) A total of 779 transcripts were identified as differentially expressed genes (DEGs; adjusted p-value cutoff < 0.05 and |log2 fold-change| > 1). Red dots in the volcano plot indicate DEGs. (C) Heatmaps show expression patterns of genes belonging to a given pathway. The Morpheus application (https://software.broadinstitute.org/morpheus/) was used to cluster genes with the hierarchical clustering method. The one-minus pearson-correlation metric was used.

**
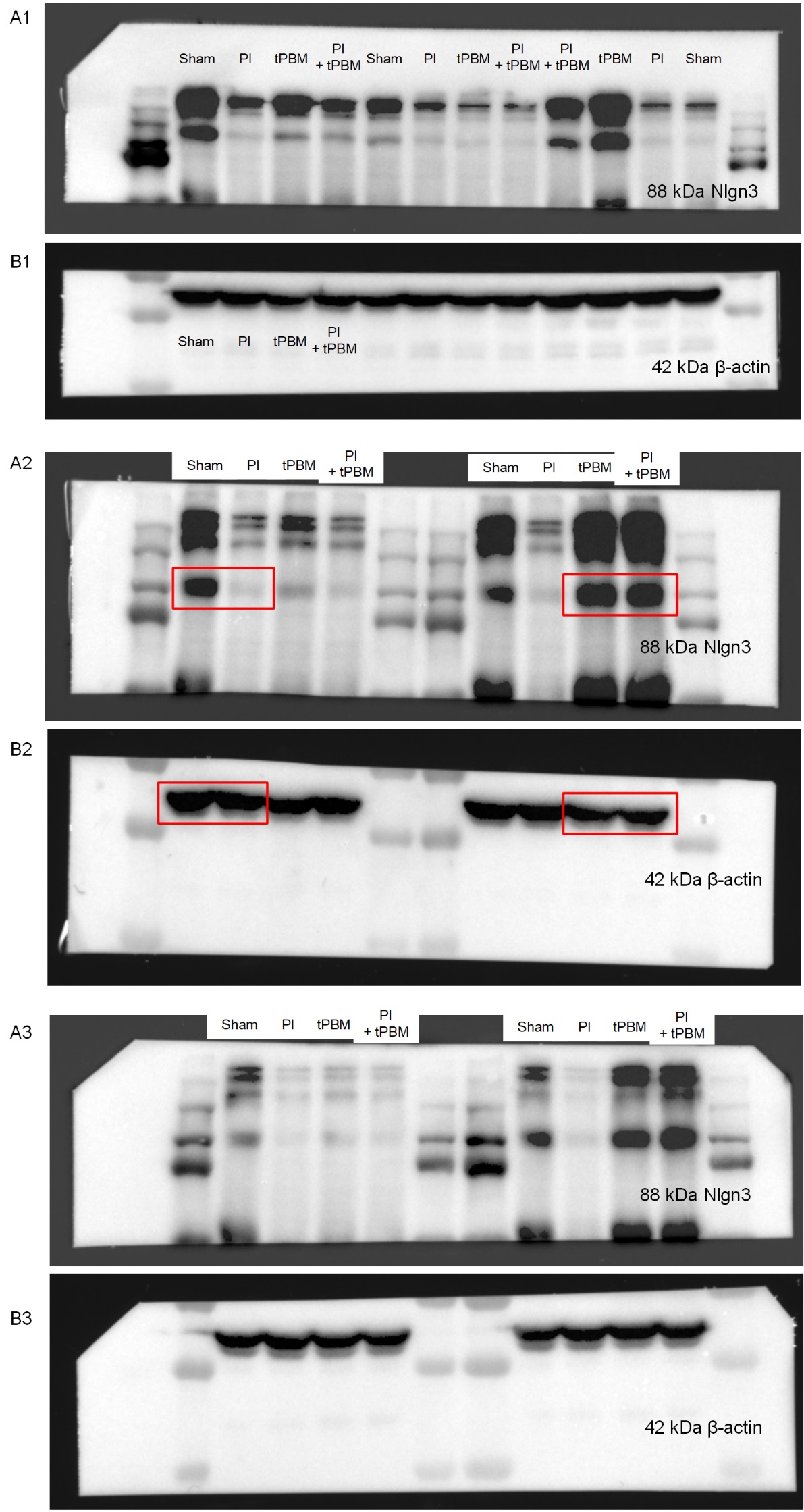
**

**Figure S7.** Full-length blots of neuroligin-3 antibody. (A1-3) Nlgn3, (B1-3) β-actin of Nlgn3

**
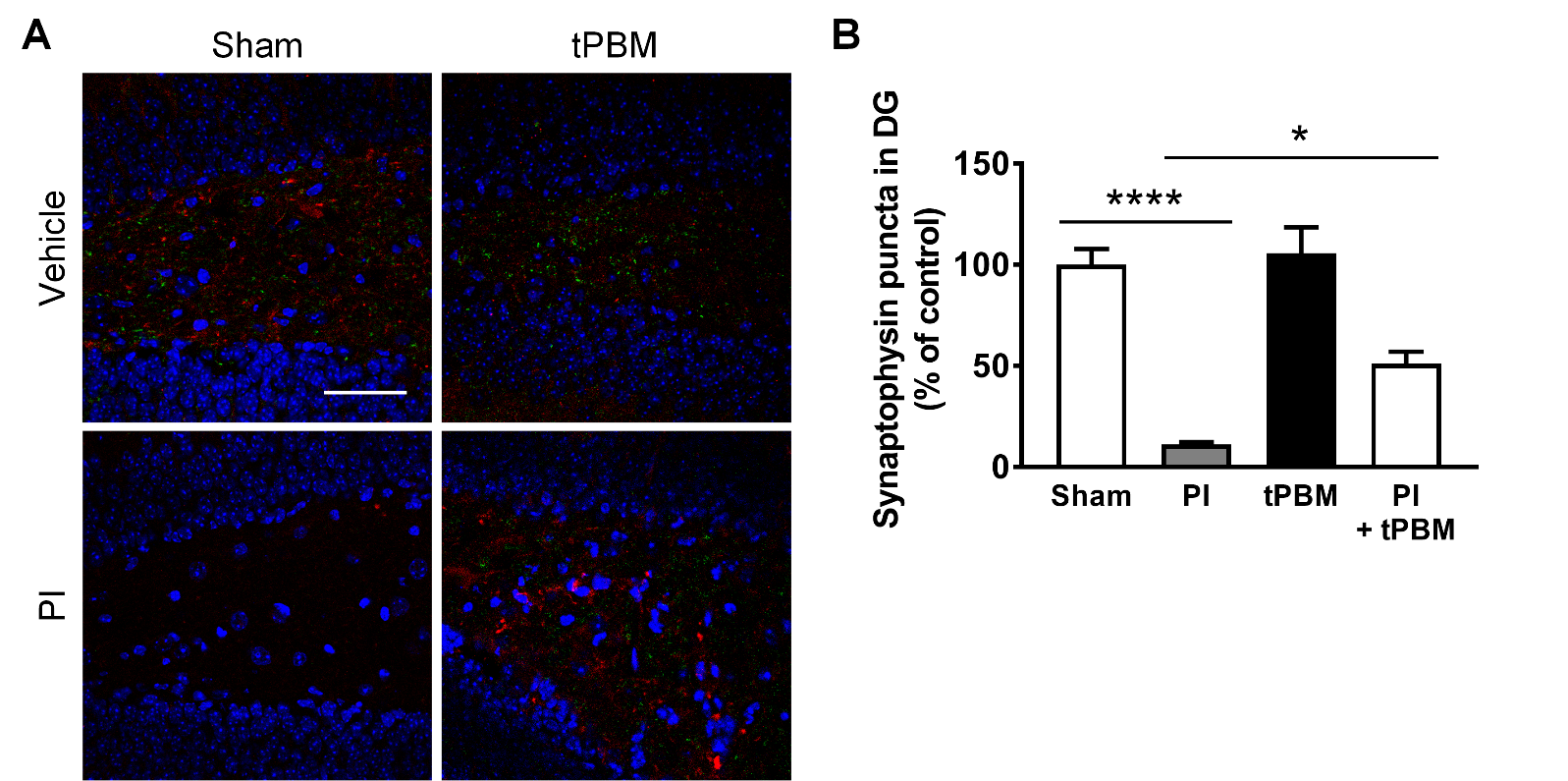
**

**Figure S8.** The evaluation of pre-synaptic terminals in the DG of hippocampus on pilocarpine-induced excitotoxicity mouse using Synaptophysin puncta analysis. Photobiomodulation increases Synaptophysin puncta in DG (scale bar = 50 μm). Data are expressed as the mean ± SEM; *p < 0.05, ****p < 0.0001 relative to PI (ANOVA with the Bonferroni test).

**
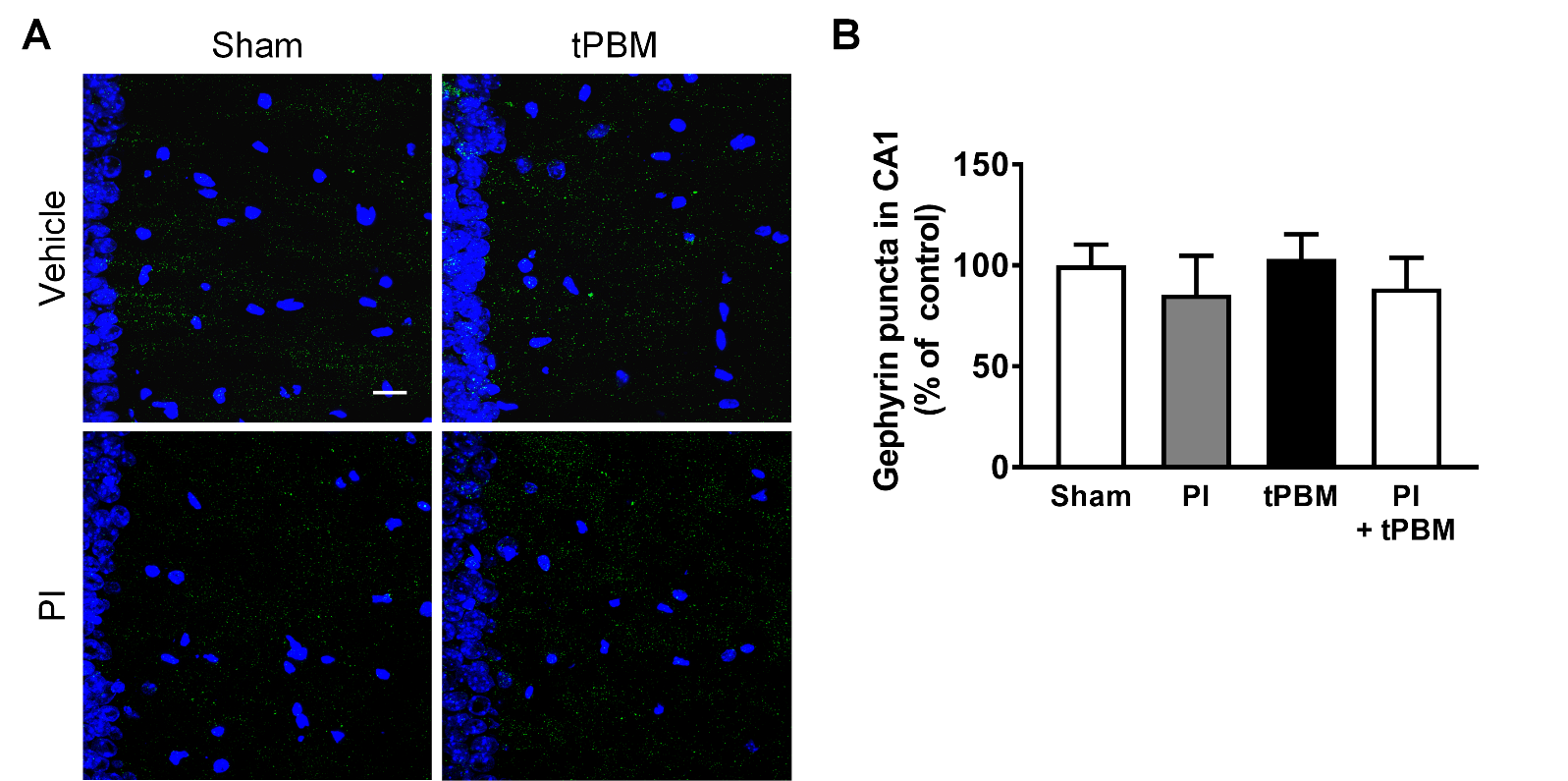
**

**Figure S9.** The evaluation of inhibitory synapses in the CA1 of hippocampus on pilocarpine-induced excitotoxicity mouse using Gephyrin puncta analysis. Photobiomodulation does not alter Gephyrin puncta in CA1 (scale bar = 20 μm).


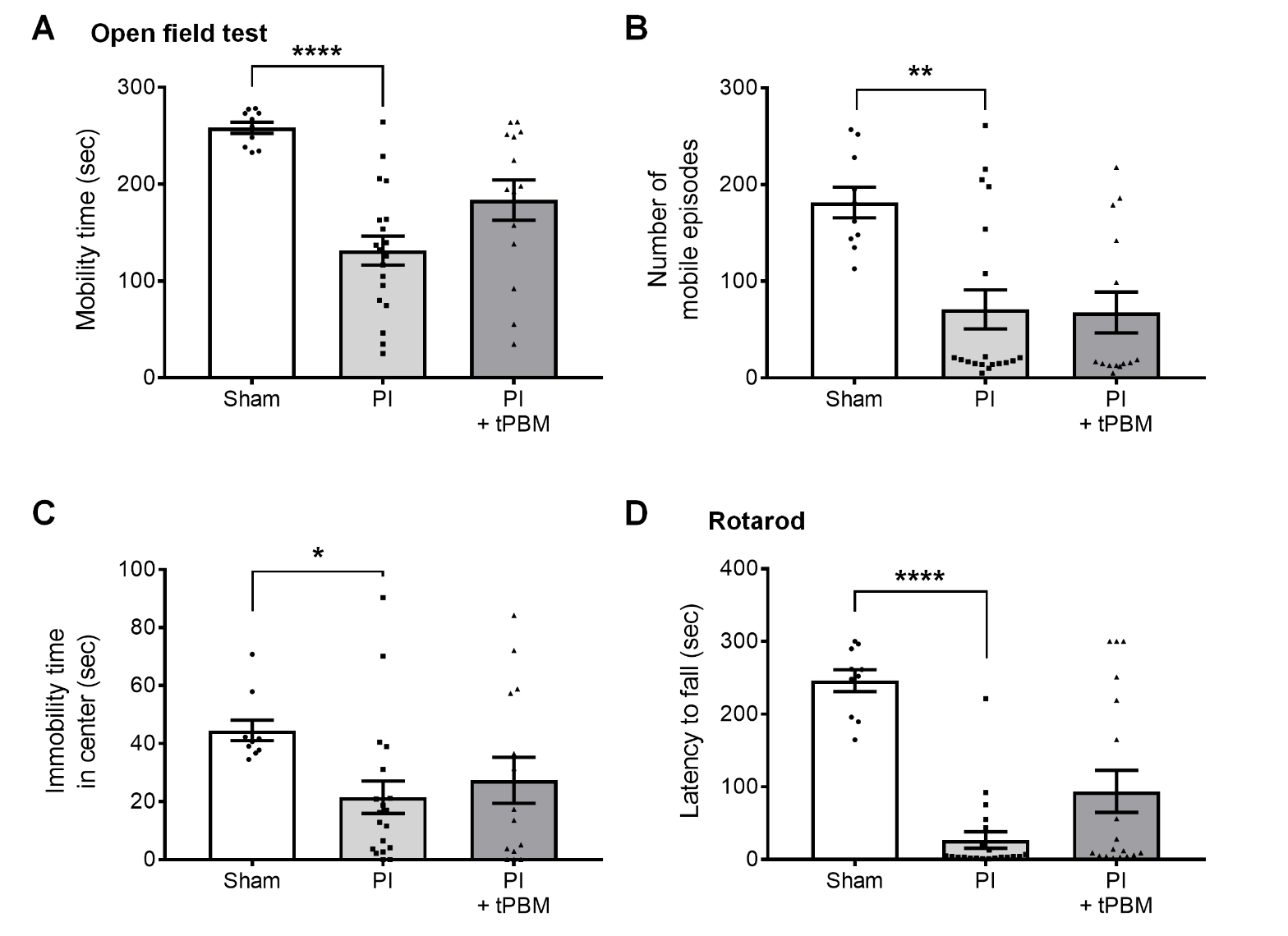


**Figure S10.** OFT results of the other parameter and Rotarod test. (A) OFT; mobility time (B) OFT; number of mobile episodes (C) OFT; immobility time in center (D) Rotarod test. Data are expressed as the mean ± SEM; *p < 0.05, **p < 0.01, ****p < 0.0001 relative to PI (ANOVA with the Bonferroni test).

**Additional videos**

Video S1. Stage 1 and 2 of modified Racine scale. <http://gofile.me/56Nwu/LwLigVeWN>

Video S2. Stage 3 of modified Racine scale. <http://gofile.me/56Nwu/uD0DeNmem>

Video S3. Stage 4 of modified Racine scale. <http://gofile.me/56Nwu/9T2uO9Gm0>

Video S4. Stage 5 of modified Racine scale. <http://gofile.me/56Nwu/fzHq2tk8e>

Video S5. Stage 6 of modified Racine scale. <http://gofile.me/56Nwu/H5crncftq>

Video S6. Acute seizure after stage 6; status epilepticus. <http://gofile.me/56Nwu/gvf4ks9u4>
